# Effects of Fermented Palm Kernel Meal on Lactation Performance, Rumen Fermentation, Rumen Microbiota, and Rumen Metabolomic Profiles in Holstein Dairy Cows

**DOI:** 10.3390/vetsci13080777

**Published:** 2026-08-03

**Authors:** Xianglong Zhang, Xitong Guan, Jiahui Cao, Yuxuan Yan, Yueyang Zhao, Hongxiang Mao, Lizhou Ma, Lingling Huang, Xiangfang Tang, Shunjin Jiang, Yang Li

**Affiliations:** 1 College of Animal Science and Technology, Northeast Agricultural University, Harbin 150030, China; zxl16695190908@163.com (X.Z.); gxt20010827@163.com (X.G.); 17612845180@163.com (J.C.); 18800496696@163.com (Y.Y.); 18246893818@163.com (Y.Z.); maohongxiang@cn.wilmar-intl.com (H.M.); 2 Wilmar (Shanghai) Biotechnology Research and Development Center Co., Ltd., Shanghai 200137, China; malizhou@cn.wilmar-intl.com (L.M.); huanglingling@cn.wilmar-intl.com (L.H.); 3 State Key Laboratory of Animal Nutrition, Institute of Animal Science, Chinese Academy of Agricultural Sciences, Beijing 100193, China; tangxiangfang@caas.cn; 4 Guangdong Rongda Biology Co., Ltd., Qingyuan 511510, China; shunjin2000@sina.com

**Keywords:** fermented palm kernel meal, β-mannan degradation, nutrient digestibility, pro-inflammatory cytokine, solid-state fermentation

## Abstract

This study evaluated the impact of fermented palm kernel meal (FPKM) on the lactation performance, blood biochemical indices, rumen microbiota, and metabolic functions of Holstein dairy cows, aiming to enhance the nutritional value of palm kernel meal (PKM) through solid-state fermentation. A 3 × 3 Latin square design was used, involving 12 multiparous Holstein cows (parity = 3; body weight = 625 ± 25.8 kg; days in milk = 103 ± 19.6 day(s); milk yield = 32.6 ± 1.58 kg/d) over three 28-day periods. Cows were randomly assigned to three isocaloric and isonitrogenous diets: a basal diet with wheat bran (WB group), a diet with wheat bran replaced by PKM (PKM group), and a diet with wheat bran replaced by FPKM (FPKM group). Solid-state fermentation improved PKM’s nutritional profile by reducing fiber and β-mannan content while increasing protein availability and ruminal degradability. Compared to the WB group, the PKM group showed lower dry matter intake, milk yield, and nutrient digestibility. In contrast, the FPKM group had higher DMI and milk yield than the PKM group, improved nutrient digestibility, and the highest energy-corrected milk yield due to increased milk protein and lactose production. The FPKM group also had higher concentrations of total volatile fatty acids, propionate, acetate, and microbial protein synthesis than the PKM group. Pro-inflammatory cytokines (tumor necrosis factor-α, interleukin-8) were elevated in the PKM group but were reduced to levels similar to the WB group in the FPKM group. Plasma immunoglobulin G levels were higher in both the FPKM and WB groups compared to the PKM group. The FPKM group also showed increased relative abundances of *Prevotella*, *Fibrobacterota*, and *Verrucomicrobiota*, while *Bacillota* and *Ruminococcus* were reduced compared to the WB group. Metabolomic profiling revealed that FPKM upregulated energy metabolism and inflammation-related pathways, increasing metabolites such as riboflavin and adenine and decreasing succinic acid and guanine compared to the PKM group. In conclusion, FPKM improved the feeding value of PKM-based material and showed more favorable responses than PKM, with generally comparable responses to WB, supporting its potential as an alternative feed ingredient for lactating dairy cows.

## Simple Summary

Palm kernel meal is produced in large amounts during palm oil processing and could be a useful feed resource for dairy cows. However, its direct use is limited by its high fiber content and by β-mannan, a carbohydrate that can reduce nutrient use and may affect animal health. Fermentation may help overcome these limitations by breaking down part of the fiber and improving the nutritional value of the material. In this study, we compared wheat bran, unfermented palm kernel meal, and fermented palm kernel meal in the diets of lactating Holstein cows. Cows fed unfermented palm kernel meal showed lower feed intake, milk yield, and nutrient digestibility, and they also had higher levels of some inflammatory markers. By contrast, cows fed fermented palm kernel meal had better feed intake, milk production, nutrient digestibility, and rumen fermentation than those fed unfermented palm kernel meal. The fermented product also influenced rumen bacteria and metabolites, which may help explain the improved responses. These results suggest that fermented palm kernel meal could be a practical alternative feed ingredient for lactating dairy cows, especially when wheat bran is costly or in limited supply.

## 1. Introduction

Amid global feed resource constraints and increasing competition between human food and animal feed demands, the exploration of alternative feed resources and the optimization of agricultural by-product use are critical for the sustainable advancement of the livestock industry. Palm kernel meal (PKM) is a by-product of palm fruit processing. As a widely cultivated oil crop in tropical regions, oil palm has a significantly higher oil yield per unit area than other oil crops [1]. According to 2023 data from the Food and Agriculture Organization (FAO), the annual global production of PKM has reached 9 million metric tons. Although PKM is rich in protein and fiber, it is often discarded or sold at a low price due to its high fiber content and the presence of anti-nutritional factors such as β-mannan [2]. Discarded PKM releases large amounts of methane during natural decomposition, exacerbating greenhouse gas emissions; moreover, leachate from long-term storage can pollute soil and water, further increasing environmental pressure [3]. Therefore, promoting the high-value utilization of PKM is of great significance for achieving sustainable feed resource utilization and reducing environmental impacts.

Fermentation technology, a core method for developing unconventional feed resources, shows considerable potential for improving feed quality. Through solid-state fermentation, PKM can be converted into fermented palm kernel meal (FPKM). The high concentrations of structural carbohydrates and β-mannan in untreated PKM can restrict microbial access to nutrients and reduce its digestibility and feeding value in ruminants. During fermentation, microbial enzymatic activity can partially disrupt the fiber matrix, hydrolyze β-mannan, and increase the availability of soluble and readily degradable nutrients [2,4,5]. Consequently, FPKM generally contains less crude fiber and β-mannan than PKM and may have greater ruminal degradability and nutrient availability, thereby increasing the practical value of this abundant agro-industrial by-product as an alternative feed resource for ruminants [4,5]. With a crude protein content of approximately 16.6% and a higher fat content than corn and soybean meal, FPKM can be classified as a potential energy feed. WB, a major by-product of wheat processing, is a vital component of ruminant diets due to its availability and high nutritional value [6]. However, the supply of WB is uncertain and its price is volatile amid growing global food demand and fluctuating feed markets, necessitating the search for cost-effective and sustainable alternatives [7]. FPKM, with its stable supply and lower production costs, presents a promising and economically viable alternative to WB, potentially reducing feed formulation costs while maintaining or even enhancing animal performance [8]. It helps to explore the utilization channels of PKM to reduce environmental pollution, showing good prospects for promotion and application value.

β-mannan is a major anti-nutritional factor in PKM and contributes to its lower feeding value by reducing nutrient availability and digestive efficiency, with potential adverse effects on gut and immune homeostasis [9,10]. Although the rumen microbial community, including bacteria and fungi capable of producing β-mannanase, can partially degrade β-mannan, this degradation may be insufficient to fully overcome the nutritional constraints associated with high-β-mannan and high-fiber PKM [11]. Microbial fermentation can hydrolyze β-mannan and other fibrous components in PKM [12], thereby improving the nutritional profile of FPKM and providing a basis for its use as a potential WB substitute in ruminant diets.

The application in monogastric animals has shown that FPKM can optimize the intestinal microbiota structure and improve growth performance and nutrient digestibility in broilers and piglets [8,13]. In beef cattle diets, replacing soybean meal with 3% FPKM did not adversely affect dry matter intake or digestibility and improved feed conversion efficiency, rumen microbial composition, and metabolic functions [14]. From the perspective of ruminant production, improving the feeding value of PKM through fermentation could broaden the utilization of agro-industrial by-products and reduce reliance on conventional feed ingredients. This strategy may also increase flexibility in dairy diet formulation and contribute to more resource-efficient and sustainable production. Based on this background, we hypothesized that solid-state fermentation would transform palm kernel meal (PKM) into a high-quality feed (FPKM) by degrading its fibrous structure and β-mannan content. We postulated that substituting wheat bran (WB) with FPKM would improve lactation performance in dairy cows through a coordinated mechanism involving enhanced ruminal fermentation (e.g., increased volatile fatty acids and microbial protein), a reduction in β-mannan-induced systemic inflammation, and a beneficial shift in the rumen microbiome and metabolome towards an energy-efficient and anti-inflammatory phenotype. The comprehensive measurements of production parameters, rumen function, blood biomarkers, and microbial omics in this study were designed to test this integrated hypothesis.

## 2. Materials and Methods

### 2.1. Preparation for FPKM

The experimental microbial consortium, consisting of *Lactobacillus acidophilus* (CGMCC 25532), *Saccharomyces cerevisiae* (CGMCC 6183), and *Bacillus subtilis* (CGMCC 7641) in a ratio of 1:3:4.5, was provided by the Ruminant Nutrition Laboratory at Yihai Kerry Arawana Holdings Co., Ltd., Shanghai R&D Center (Shanghai, China). This ratio was selected based on preliminary process optimization and the complementary roles of the three microorganisms in the PKM-based solid-state fermentation system. *Lactobacillus acidophilus* was used to promote acidification and microbial stability, *Saccharomyces cerevisiae* was included to support fermentation activity and improve substrate palatability through yeast-derived metabolites, and *Bacillus subtilis* was used as the main enzyme-producing microorganism to promote the degradation of fiber, β-mannan, and protein fractions. The raw materials, including PKM and soybean molasses, were sourced from Yihai Jinlongyu Food Group Co., Ltd. (Shanghai, China). All fermentation processes were conducted under identical conditions at the aforementioned facility.

Following heat sterilization of PKM at 121 °C for 30 min, the processed substrate was mixed with wheat middlings (8% of PKM dry weight), soybean molasses (3% of PKM dry weight), municipal tap water, and microbial inoculum (≥1.2 × 10^10^ CFU/mL) to achieve a final moisture content of approximately 40% (*w*/*w*). The inoculation rate was adjusted to 0.5% of PKM dry weight. The mixture was then loaded into a specialized fermentation vessel supplied by Nanjing Runzhe Biotechnology Equipment Co., Ltd. (Nanjing, China), where mechanical stirring ensured homogeneous mixing before fermentation. The vessel was then sealed, and anaerobic solid-state fermentation was conducted for 96 h at 33 ± 1 °C. After fermentation, the final product (FPKM) was dried at 55 °C to approximately 10% moisture, milled, and packaged. Each batch of the finished product was sampled for quality control. Representative subsamples were ground and sieved through a 1 mm mesh prior to analysis. Nutrient composition and β-mannan content were determined following the procedures described in the main text to confirm compliance with the targeted nutritional specifications. The coefficient of variation (CV) for major nutritional parameters among batches was maintained below 5%.

### 2.2. Animals, Experimental Design, and Diets

This research protocol was approved by the Institutional Animal Care Committee of Northeast Agricultural University (Ethical Clearance No: NEAUEC20240259; Harbin, China) and was conducted at Harbin Wondersun Dairy Cattle Breeding Co., Ltd., located in Duiqingshan Town, Songbei District, Harbin, Heilongjiang Province, China, from August to October 2024. The cows were housed in individual stalls in a semi-open barn equipped with mechanical ventilation, fans, and sprinklers. Twelve healthy lactating Holstein cows (parity = 3), with a mean (±SD) body weight of 625 ± 25.8 kg, days in milk of 108 ± 19.6 d, and daily milk yield of 32.6 ± 1.58 kg/d, were randomly allocated to four replicated 3 × 3 Latin squares, resulting in four cows per dietary treatment in each experimental period. Dry matter intake and lactation performance were recorded for all cows, yielding 12 cow–period observations per treatment across the three periods (*n* = 12). For ruminal and fecal fermentation, apparent total-tract nutrient digestibility, plasma biomarkers, rumen microbiota, and rumen metabolomics, two cows were randomly selected from each treatment in each period, yielding six cow–period observations or samples per treatment (*n* = 6). Each period began with a 21-day feed adaptation period prior to a 7-day intensive sampling window for data acquisition.

The study employed a randomized allocation of lactating cows into three distinct feeding regimens: (1) WB group fed a basal diet containing wheat bran; (2) PKM group with complete wheat bran substitution using palm kernel meal; (3) FPKM group receiving fully fermented PKM as replacement. The three diets differed in the inclusion of WB, PKM, or FPKM at 5.86% of dietary dry matter (DM), while the inclusion rates of the remaining ingredients were unchanged. The diets were formulated using the Cornell–Penn–Miner Dairy Model (CPM Dairy, version 3.0.10) to provide similar crude protein and net energy for lactation concentrations. Because WB, PKM, and FPKM differed in chemical composition, small differences remained among the diets in neutral detergent fiber (NDF), acid detergent fiber (ADF), non-fibrous carbohydrate (NFC), physically effective neutral detergent fiber (peNDF), starch, and ether extract (EE) (Table 1). The peNDF was determined according to Mertens [15] using the following equation:

peNDF (% of DM) = NDF (% of DM) × pef,

where NDF is the analyzed NDF concentration of the total mixed ration (TMR), and pef is the physical effectiveness factor derived from the particle-size distribution of the total mixed ration measured using the Penn State Particle Separator (PSPS). The monthly mean temperature and relative humidity inside the barn were 24.1 °C and 81% in August and 20.8 °C and 70% in September, respectively. The corresponding temperature–humidity index values, estimated from the monthly mean temperature and relative humidity, were 73.54 and 67.52, respectively. Mechanical ventilation, fans and sprinklers were used to alleviate heat load. Cows were milked twice daily at 07:00 and 19:00. Fresh total mixed rations were prepared and dispensed twice daily (08:00 and 20:00), with daily feed adjustments ensuring that the refusal rates were maintained at approximately 3–5%. Continuous access to clean drinking water was provided throughout the experimental duration.

The chemical compositions of the WB, PKM, and FPKM used in the experimental diets are presented in Table 2. These values are provided as a descriptive characterization of the feed ingredients.

### 2.3. Data Collection and Sampling

#### 2.3.1. Feeds, Feces, and Milk

Throughout the 22ndth to 28th day period within each experimental stage, TMR consumption and leftover amounts were documented each day across seven successive days to calculate dry matter consumption. During the concluding three-day interval of every phase, 500 g representative specimens of dietary constituents, unconsumed portions, and TMR were accumulated, stored at −20 °C prior to being thoroughly blended per bovine subject and experimental cycle. These materials were subjected to 55 °C oven dehydration for 48 h followed by pulverization using 1 mm sieves.

Between days 23–25, approximately 500 g fecal samples were collected via rectal sampling at five scheduled intervals daily (08:00, 11:00, 14:00, 17:00, 20:00), generating aggregated samples per ruminant. These samples underwent identical processing to nutritional specimens for digestibility analysis, with aliquots snap-frozen in liquid nitrogen for later microbiota determination.

Milk yield monitoring occurred daily from day 22 through day 28 using computerized milking equipment. During days 22–25, pooled milk samples (50 mL per milking event) were collected, preserved through the addition of potassium dichromate to achieve a 0.1% final concentration, and refrigerated at 4 °C before conducting milk component evaluations.

#### 2.3.2. Blood and Ruminal Fluid

During experimental days 26–28, caudal venous blood specimens were collected three hours before morning feeding (06:00 h). Plasma separation involved centrifugation at 3000× *g* for 15 min under 4 °C conditions, with subsequent cryopreservation at −80 °C. Parallel rumen content sampling was conducted using an ororuminal probe inserted approximately 150 cm into the rumen to reach the dorsal sac. To mitigate salivary contamination, the initial 200 mL of retrieved material was discarded prior to sample collection. Immediate pH measurements were recorded using a handheld pH analyzer (PHS-3C, Shanghai INESA Scientific Instrument Co., Ltd., Shanghai, China). Preservation methodologies included: (1) treatment with 10% (*w*/*v*) metaphosphoric acid for subsequent volatile fatty acids (VFA) analysis; (2) acidification with 0.5 mol/L sulfuric acid for ammonia nitrogen (NH_3_-N) determination, with both treated samples maintained at −20 °C for storage.

Rumen samples were centrifuged at 12,000× *g* for 10 min at 4 °C. The resulting supernatant was immediately stored at −80 °C for subsequent metabolomic analysis. The remaining pellet, representing the microbial fraction enriched from rumen fluid, was washed three times with PBS and stored at −80 °C for DNA extraction and microbiota analysis.

#### 2.3.3. Feed Nutrient Analysis Procedures

Quantification of DM, ash, crude protein (CP), starch, and ether extract (EE) were determined in compliance with AOAC standard procedures [18]. The evaluation of thermally resistant α-amylase-treated fibrous components including NDF, ADF, and acid detergent lignin (ADL) followed the analytical approach outlined by [19]. Protein compartmentalization studies involved measuring detergent-resistant protein fractions (neutral detergent insoluble crude protein [NDICP] and acid detergent insoluble crude protein [ADICP]) using the research group’s standardized procedures. The soluble protein (SP) and non-conventional nitrogen (NPN) were assessed through specialized techniques following the experimental framework established by [20]. NFC content was calculated using the formula: NFC (% DM) = 100 − [Ash (% DM) + CP (% DM) + EE (% DM) + NDF (% DM)], where DM represents dry matter basis. This computational approach subtracts the combined proportions of structural fiber, protein, lipid, and mineral components from total dry matter to quantify soluble carbohydrate fractions.

#### 2.3.4. Nutrient Digestibility and Milk Composition

Apparent total-tract digestibility of DM, CP, NDF, and ADF was estimated using indigestible neutral detergent fiber (iNDF) as an internal marker [21]. The iNDF concentrations in the TMR, refusals, and feces were determined as the residual NDF after 288 h of in situ ruminal incubation [22]. Daily iNDF intake and fecal DM output were calculated as follows:

iNDF intake (kg/d) = (DM offered × iNDF_TMR) − (DM refusals × iNDF_refusals),

Fecal DM output (kg/d) = iNDF intake/iNDF_feces

Dry matter intake and apparent DM digestibility were calculated as follows

$$DMI={DM}_{offered}-{DM}_{refusals}$$

Apparent DM digestibility (%) = [1 − (fecal DM output/DMI)] × 100

For CP, NDF, and ADF, nutrient intake, fecal nutrient output, and apparent total-tract digestibility were calculated according to the conventional intake–output method [23] as follows:

*X*_intake_ (kg/d) = (DM_offered_ × *X*_TMR_) − (DM_refusals_ × *X*_refusals_),

Fecal *X*_output_ (kg/d) = fecal DM output × *X*_feces_,

Apparent digestibility of *X* (%) = [1 − (fecal *X*_output_/*X*_intake_)] × 100

where *X* represents CP, NDF, or ADF; DM_offered_ and DM_refusals_ represent the daily amounts of TMR offered and refused on a DM basis, respectively; iNDF_TMR_, iNDF_refusals_, and iNDF_feces_ represent the iNDF concentrations in the TMR, refusals, and feces, respectively; and *X*_TMR_, *X*_refusals_, and *X*_feces_ represent the corresponding nutrient concentrations. DMI represents daily dry matter intake. All concentration terms were expressed as decimal proportions of DM in the calculations. Comprehensive milk component evaluation was conducted at Heilongjiang Academy of Agricultural Reclamation (Harbin, China) employing a MilkoScan FT1 multi-channel spectrophotometric analyzer (Foss Electric, Hillerød, Denmark) for precise measurement of protein constituents, lipid profiles, carbohydrate fractions, milk urea nitrogen (MUN), and somatic cell counts (SCC).

#### 2.3.5. Plasma, Ruminal Fermentation, and Ruminal Degradation Kinetics

Validated commercial diagnostic kits were used to evaluate plasma metabolic indicators, oxidative stress biomarkers, and immunological markers. Plasma non-esterified fatty acids (NEFA), β-hydroxybutyrate (BHBA), glucose (GLU), complement component C3, malondialdehyde (MDA), total antioxidant capacity (T-AOC), and total superoxide dismutase (T-SOD) activity were determined using commercial assay kits from Jiancheng Bioengineering Institute (Nanjing, China), and the analytical reagents were sourced from Jiangsu Addison Biotechnology Co., Ltd. (Yancheng, China), according to the manufacturers’ instructions. Immunoglobulin subtypes (IgA, IgM, and IgG) and pro-inflammatory cytokines (TNF-α, IL-1β, IL-6, IL-8) were measured using enzyme-linked immunosorbent assay (ELISA) kits from Jiangsu Meimian Industrial Co., Ltd. (Yancheng, China) on a Tecan Infinite 200 Pro microplate reader (Tecan Group Ltd., Männedorf, Switzerland), following the manufacturers’ instructions.

VFA profiles were determined via gas chromatographic analysis (GC-8A instrument, Shimadzu Corp., Kyoto, Japan) equipped with a DB-WAX capillary column (Agilent Technologies, Santa Clara, CA, USA; specifications: 30 m length × 0.32 mm internal diameter × 0.25 μm film thickness) and a flame ionization detector, based on standardized protocols. Ammonia nitrogen concentrations were determined using the phenol-hypochlorite spectrophotometric method [24] whereas rumen degradation parameters were assessed following documented procedures [25]. 

Three rumen-cannulated Holstein cows (mean body weight 653 ± 27.8 kg) were housed individually and fed twice daily at 07:00 and 19:00 with ad libitum water access. The total mixed ration formulation contained 589.3 g/kg concentrated feed mixture, 313.1 g/kg maize silage, and 107.6 g/kg alfalfa hay on a dry matter basis. Exactly 7 g of desiccated PKM and FPKM samples were individually loaded into pre-labeled nylon mesh bags (10 × 20 cm, 50 μm pore size). Samples were anchored to 1.5 m nylon cords and placed in the rumen’s dorsal sac for predetermined periods (0, 2, 4, 8, 12, 16, 24, 36, 48 h). After removal, bags were rinsed with deionized water until runoff cleared, then oven-dried at 55 °C for 48 h to constant weight. Samples were subsequently conditioned in a humidity chamber (24 h) before. Residual samples were finely ground using a 1 mm sieve for subsequent analysis of nutrient composition including dry matter, crude protein, and neutral detergent fiber content. Ruminal degradation kinetics were fitted using the exponential model described by Ørskov and McDonald [26]:

*p* = *a* + *b*(1 − *e^−ct^*),

where *p* is the proportion of the sample degraded after incubation time t (%); *a* is the rapidly degradable fraction (%); *b* is the insoluble but potentially degradable fraction (%); *c* is the fractional degradation rate constant of fraction *b* (h^−1^); t is the ruminal incubation time (h); and e is the base of the natural logarithm. Effective ruminal degradability (ED) was calculated as:

*ED* = *a* + *bc*/(*c* + *k_p_*),

where *ED* is the effective ruminal degradability (%), and *k_p_* is the assumed fractional ruminal passage rate, which was set at 0.06 h^−1^. The values of *c* presented as %/h in Table 3 were converted to h^−1^ before calculating *ED*. β-mannan levels in temporal samples collected at 24 h and 48 h intervals were quantified following the GB/T 6006-2024 standard methodology [17], with microbial degradation patterns evaluated through the pioneering kinetic framework developed by [26].

### 2.4. Bacterial Communities

For microbiome and metabolome analyses, a stratified random sampling approach was employed. Rumen fluid was collected from 2 cows randomly selected from each dietary treatment within each experimental period, resulting in a total of 6 biological replicates per treatment across the 3 experimental periods (which contained four cows each). This design resulted in a total of six biological replicates per treatment across the three periods, which were used to ensure robust statistical analysis of community and metabolic profiles. Rumen fluid samples were stored at −80 °C for subsequent 16S rRNA gene sequencing analysis via PCR amplification. Microbial genomic DNA was extracted with the E.Z.N.A.^®^ Soil DNA Extraction Kit (Omega Bio-tek, Norcross, GA, USA), with initial DNA quality verification performed through 1% agarose gel electrophoresis (30-min run at 120 V). Quantitative DNA assessment and purity verification were performed using a NanoDrop 2000 UV–Vis spectrophotometer (Thermo Fisher Scientific, Waltham, MA, USA), ensuring absorbance ratios (A_260_/A_280_) remained within 1.8–2.0. The V3-V4 hypervariable regions of bacterial 16S rRNA genes were amplified through PCR with specific primers 338F (5′-ACTCCTACGGGAGGCAG-3′) and 806R (5′-GGACTACGGGTWTCTAAT-3′). The 25 μL reaction system contained 12.5 μL of 2 × Taq PCR MasterMix solution, 1 μL each of forward/reverse primers (10 μM concentration), 2 μL genomic DNA template (50 ng/μL), and 8.5 μL sterile ultrapure water. Thermocycling conditions were programmed as follows: primary denaturation at 95 °C for 5 min.

The amplification procedure involved 30 cycles of thermal cycling initiated by a preliminary denaturation step (95 °C, 5 min). Each cycle consisted of three phases: DNA strand separation (95 °C, 30 s), primer hybridization (55 °C, 30 s), and polymerase-mediated elongation (72 °C, 45 s), culminating in a final elongation stage (72 °C, 7 min). Amplification products underwent electrophoretic separation for 40 min at 120 V using a 2% agarose matrix, followed by purification with the AxyPrep DNA Gel Extraction Kit (Axygen Biosciences, Union City, CA, USA) according to standardized protocols. Post-purification verification was achieved through repeat electrophoresis analysis. Processed DNA samples were accurately measured using the Quantus^™^ Fluorometric System (Promega, Madison, WI, USA) to determine nucleic acid concentrations for subsequent experimental procedures.

The experimental workflow involved library preparation services provided by Majorbio Bio-Pharm Technology Co., Ltd. (Shanghai, China), employing the NEXTFLEX Rapid DNA-Seq Kit manufactured by PerkinElmer (Waltham, MA, USA). Subsequent high-throughput sequencing employed the Illumina MiSeq PE300 system (Illumina, San Diego, CA, USA) for data generation. Initial sequence data were subjected to rigorous quality control procedures using Fastp software (v0.20.0) with strict filtering criteria (minimum quality threshold of Q20 and read length truncation at 150 bp). Paired-end read merging was carried out via the Flash algorithm (v1.2.11) with a 10 bp overlap requirement. Taxonomic analysis involved clustering quality-filtered sequences into operational taxonomic units (OTUs) applying a 97% sequence similarity threshold through the Uparse workflow (v7.0.1090), with chimeric sequences detected and removed through embedded UCHIME algorithm functionality.

The taxonomic assignment of OTU representative sequences was performed through the RDP classifier (v2.13) utilizing the Silva 16S rRNA reference database (Release 138), with a confidence cutoff set at ≥0.7. These sequencing datasets have been archived in the NCBI Sequence Read Archive under the accession identifier PRJNA1297090.

### 2.5. Non-Targeted Metabolomics Analysis

A composite quality control (QC) sample was prepared by pooling equal 10 μL aliquots from all 18 rumen fluid samples used for metabolomic analysis, representing 2 cows per treatment in each period across 3 dietary treatments and 3 experimental periods (*n* = 6 per treatment). Metabolite profiling was performed using a Waters Acquity I-Class PLUS ultra-high-performance liquid chromatography system coupled to a Xevo G2-XS quadrupole time-of-flight mass spectrometer (Waters, Milford, MA, USA). Separation occurred on an HSS T3 analytical column (2.1 × 100 mm, 1.8 μm particle size) kept at 40 °C with a fixed flow rate of 0.3 mL/min. The elution program progressed through multiple stages: initial maintenance of 95% solvent A (0.1% formic acid in water) over 0–2 min, linear transition to 5% A by 10 min, constant 5% A from 10–12 min, re-equilibration at 95% A between 12–14 min, and column stabilization from 14–16 min. Analyses employed 1 μL sample injections with alternating positive/negative ionization modes. Mobile phase components included: (A) aqueous 0.1% formic acid solution and (B) acetonitrile containing 0.1% formic acid.

The analytical workflow was managed using the MassLynx V4.2 software suite (Waters), with the Xevo G2-XS QTOF mass spectrometer configured in MSe mode for high-resolution data acquisition. Dual-channel detection employed alternating collision energies (2 V for low-energy scans versus 10–40 V ramped high-energy CID) with spectral data captured every 0.2 s. Electrospray ionization parameters featured bipolar capillary voltage (±2000 V), 30 V cone voltage, and thermal settings of 150 °C (ion source) coupled with 500 °C desolvation temperature. Gas flow optimization involved 50 L/h cone gas and 800 L/h desolvation gas flows, parameters carefully calibrated to sustain stable ionization performance throughout the experimental runs.

The analytical workflow incorporated feature detection, chromatographic alignment, and compound annotation using Progenesis QI software (v2.3, Waters Corporation). Compound characterization was carried out by cross-referencing the METLIN metabolite database (https://metlin.scripps.edu/) and Biomark’s proprietary compound repository, with mass error tolerances maintained under 100 ppm for both precursor and fragment ions.

### 2.6. Statistical Analysis

The experimental cattle were assigned following a Latin square design, with treatment groups randomized through SAS 9.4 Proc Plan’s sequence generation to mitigate location-related confounding factors. Prior to statistical evaluation, potential carryover effects were assessed within the mixed-model framework by adding the previous-period treatment as a fixed effect, and no detectable lingering impacts were observed (*p* > 0.05). Given the limited power to detect subtle carryover effects in Latin square designs, nonsignificance was not interpreted as definitive evidence of no residual effects. Consequently, the complete dataset underwent analysis through SAS 9.2’s Proc Mixed methodology within a 3 × 3 Latin square configuration, maintaining rigorous control over experimental variables throughout the analytical process.

Statistical analyses were conducted using SAS 9.4 software (SAS Institute Inc., Cary, NC, USA) with the following model specification: *Y_ijkm_ = µ + T_i_ + P_j_ + C_k_ (S_m_) + S_m_ + E_ijkm_*. In this equation, *Y_ijkm_* represents observed values, µ denotes the grand mean, *T_i_* indicates treatment fixed effects (*i* = 1–3), *P_j_* corresponds to period fixed effects (*j* = 1–3), C*_k_* signifies random cow effects *(k* = 1–12), *S_m_* represents square fixed effects (*m* = 1–4), and *E_ijkm_* accounts for residual variation. When assessing carryover, the previous-period treatment was added as a fixed effect. Multiple comparisons were evaluated through Tukey’s adjusted range tests. 

For microbial community analysis, α-diversity indices, including Chao1, Shannon, Simpson, and coverage, were calculated, with coverage used to assess the sufficiency of sequencing depth among samples. The α-diversity indices and taxonomic abundance data at the phylum and genus levels were compared using Wilcoxon rank-sum tests with Benjamini–Hochberg false discovery rate adjustment for pairwise comparisons among treatments. β-Diversity patterns were analyzed using weighted UniFrac distance matrices and visualized by principal coordinate analysis (PCoA) and partial least squares discriminant analysis (PLS-DA). Differences in microbial community structure among treatments were evaluated using analysis of similarities (ANOSIM; R-value > 0.3, *p* < 0.05).

For metabolomic analysis, data acquired in the positive and negative ionization modes were merged and normalized against quality control samples before multivariate analysis. Unsupervised principal component analysis (PCA) was employed for dimensionality reduction, and orthogonal partial least squares discriminant analysis (OPLS-DA) was used for supervised classification. Model robustness was evaluated through 200-iteration permutation testing, with acceptance criteria of R^2^Y < 0.9 and Q^2^Y > 0.5.

Metabolite differences among the WB, PKM, and FPKM groups were analyzed using one-way analysis of variance (ANOVA) to determine overall statistical significance. Fold-change values were calculated between experimental groups, and variable importance in projection (VIP) values were obtained from the cross-validated OPLS-DA model. Differential metabolites were identified using the following criteria: fold change > 1.2 or <0.83, *p* < 0.05, and VIP > 1.0. The identified differential metabolites were subsequently subjected to metabolic pathway analysis and functional annotation using the Kyoto Encyclopedia of Genes and Genomes (KEGG) database. Pathway enrichment significance was evaluated using a hypergeometric distribution test with a significance threshold of *p* < 0.05.

An exploratory Pearson correlation analysis was performed between the 10 significant differential metabolites and matched phenotypic traits, including milk yield and plasma immune and inflammatory indicators (IgG, TNF-α, IL-1β, IL-8, and IL-6). The resulting correlation matrix was visualized as a heatmap.

Statistical significance was declared at *p* ≤ 0.05, whereas 0.05 < *p* ≤ 0.10 was considered a marginal trend.

## 3. Results

### 3.1. Nutrient Composition

The three experimental diets were nutritionally balanced with equivalent caloric and protein levels (Table 1). Relative to PKM, FPKM showed lower NDF, ADF, ADL, NDICP, ADICP, ether extract, and β-mannan contents, but higher small peptide, ash, SP, NPN, and starch concentrations (Table 2). Degradation kinetics analysis showed that FPKM had higher rapidly degradable fractions (a) for DM, CP, NDF, and ADF than PKM. The effective degradability values of DM and CP were also higher in FPKM than in PKM (*p* < 0.001), and similar increases were observed for NDF (*p* = 0.002) and ADF (*p* = 0.005), whereas the slowly degradable fraction (b) was reduced in FPKM (Table 3). Ruminal incubation analyses confirmed significant β-mannan reduction in FPKM at both 24-h and 48-h intervals (*p* < 0.001) (Table 4).

### 3.2. Lactation Performance

Marked variations emerged in DMI across the experimental groups (*p* < 0.001), with the FPKM-fed animals showing the highest consumption, exceeding those in the WB and PKM groups. Lactation performance parameters, including milk production and energy-corrected milk (ECM) yield, exhibited comparable tendencies among treatments (*p* < 0.01). The PKM group displayed superior milk fat composition (*p* < 0.001), but notably lower milk protein percentages compared to WB and FPKM-fed cattle (*p* = 0.002). Notably, the FPKM regimen resulted in elevated MUN concentrations compared to other dietary interventions (*p* = 0.001) (Table 5).

### 3.3. Ruminal and Fecal Fermentation Parameters

FPKM supplementation yielded elevated ruminal NH_3_-N and microbial protein levels (*p* = 0.036, *p* = 0.002), while the PKM group showed reduced values. The FPKM group also demonstrated higher ruminal total VFA production along with heightened acetate and propionate concentrations (*p* < 0.001, *p* < 0.001, *p* = 0.002). Fecal analysis revealed that animals receiving FPKM diets showed the most pronounced fecal VFA accumulation (*p* = 0.001) (Table 6).

### 3.4. Intake and Total-Tract Apparent Digestibility

The FPKM group demonstrated the greatest consumption of DM, CP, and ADF (*p* < 0.001). When assessing total-tract apparent digestibility, the PKM group showed reduced values for DM, CP, NDF, and ADF relative to both WB and FPKM groups (*p* < 0.05). Digestibility parameters remained comparable between WB and FPKM treatments (Table 7).

### 3.5. Plasma Biomarkers

Pro-inflammatory cytokine analysis revealed elevated TNF-α concentrations in the PKM group compared to WB and FPKM groups (*p* = 0.013). A similar pattern emerged for IL-8, with PKM showing a marked increase (*p* = 0.004). Immunological assessment indicated significantly diminished IgG levels in PKM versus WB and FPKM groups (*p* = 0.032) (Table 8).

### 3.6. Microbial Analysis by Amplicon Sequencing

**Figure 1:**
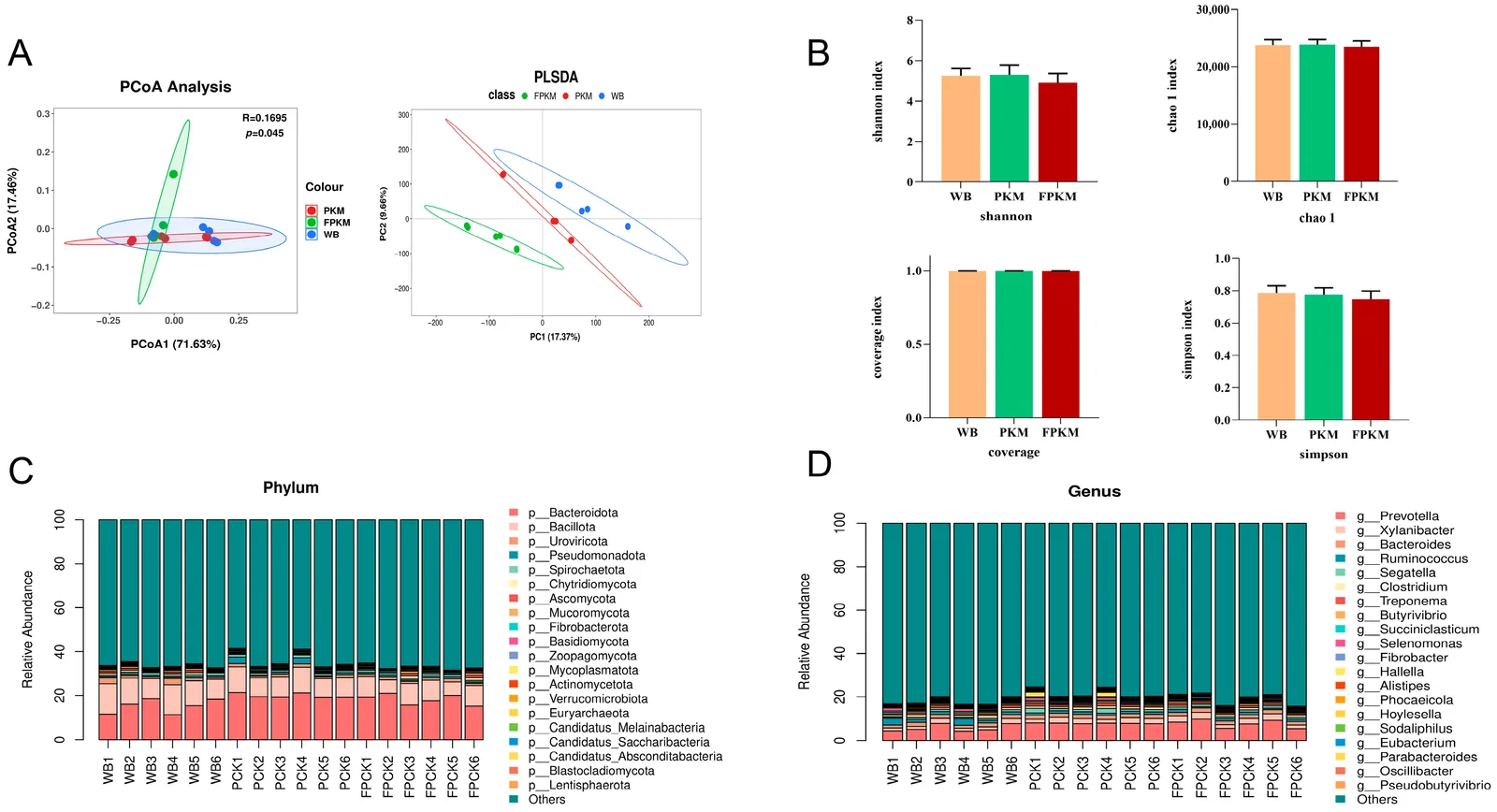
Analysis of rumen microbiota in Holstein dairy cows fed containing WB, PKM, or FPKM. (**A**) Principal coordinate analysis (PCoA) and partial least squares discriminant analysis (PLS-DA). (**B**) α-diversity indices (Chao1, Ace, Shannon, Simpson). (**C**) Relative abundance at the phylum level. (**D**) Relative abundance at the genus level. WB, A basal diet containing wheat bran; PKM, 100% replacement of wheat bran with palm kernel meal; FPKM, 100% replacement of wheat bran with fermented palm kernel meal. Each treatment included 6 biological replicates collected across the 3 experimental periods (2 cows per treatment per period).

Microbiota analysis was based on 6 biological replicates per treatment collected across the 3 experimental periods. Multivariate analyses, including PCoA and PLS-DA, revealed clear separation patterns in rumen microbiota composition across dietary interventions (Figure 1A). The α-diversity indices, including Shannon, Chao1, coverage, and Simpson, were generally comparable among the three groups (Figure 1B). At the phylum level, the relative abundance profiles differed among treatments (Figure 1C). Specifically, *Bacteroidota* (*p* = 0.008), *Saccharibacteria* (*p* = 0.045), and *Absconditabacteria* (*p* = 0.033) were more abundant in the PKM group than in the WB and FPKM groups. In contrast, compared with the WB group, the FPKM group showed greater relative abundances of *Lentisphaerota* (*p* = 0.023) and *Fibrobacterota* (*p* = 0.003), but a lower abundance of *Bacillota* (*p* = 0.019) (Table 9). At the genus level, the compositional differences are shown in Figure 1D. *Prevotella* was less abundant in the WB group than in the PKM and FPKM groups (*p* = 0.020), whereas FPKM supplementation increased *Xylanibacter* (*p* = 0.034) and reduced *Ruminococcus* (*p* = 0.011) compared with the WB group (Table 10).

### 3.7. Microbial Metabolites and KEGG Pathway Analysis

**Figure 2:**
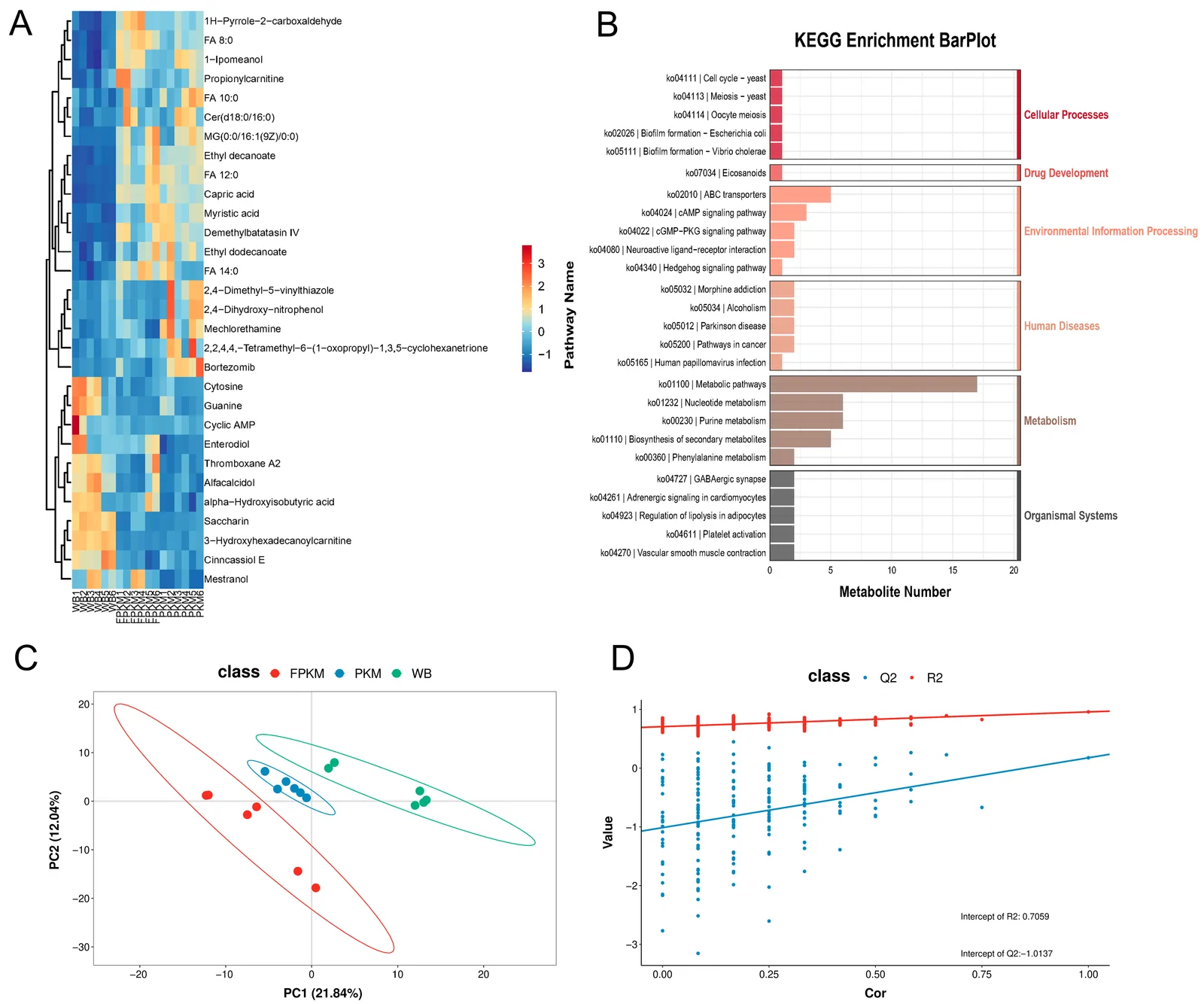
Three groups of rumen metabolite difference screening and orthogonal partial least squares discriminant analysis (OPLS-DA) of rumen metabolite differences. (**A**) Clustering heatmap of differential metabolites, the horizontal axis of the heatmap represents the samples, and the vertical axis represents the selected differential expressed metabolites; different colors represent the relative expression levels of different metabolites, with the color ranging from blue through white to red indicating the expression levels from low to high; red represents highly expressed metabolites, and blue represents lowly expressed metabolites. (**B**) Enrichment bar chart, showing the top 5 pathways of metabolites in each category of KEGG first-level classification. If a certain category is enriched to less than 5 pathways, all are displayed; if a certain first-level category does not enrich to any pathway, this category of metabolites is not displayed. (**C**) OPLS-DA score plot. (**D**) OPLS-DA model substitution test plot. WB, A basal diet containing wheat bran; PKM, 100% replacement of wheat bran with palm kernel meal; FPKM, 100% replacement of wheat bran with fermented palm kernel meal. Each treatment included 6 biological replicates collected across the 3 experimental periods (2 cows per treatment per period).

**Figure 3:**
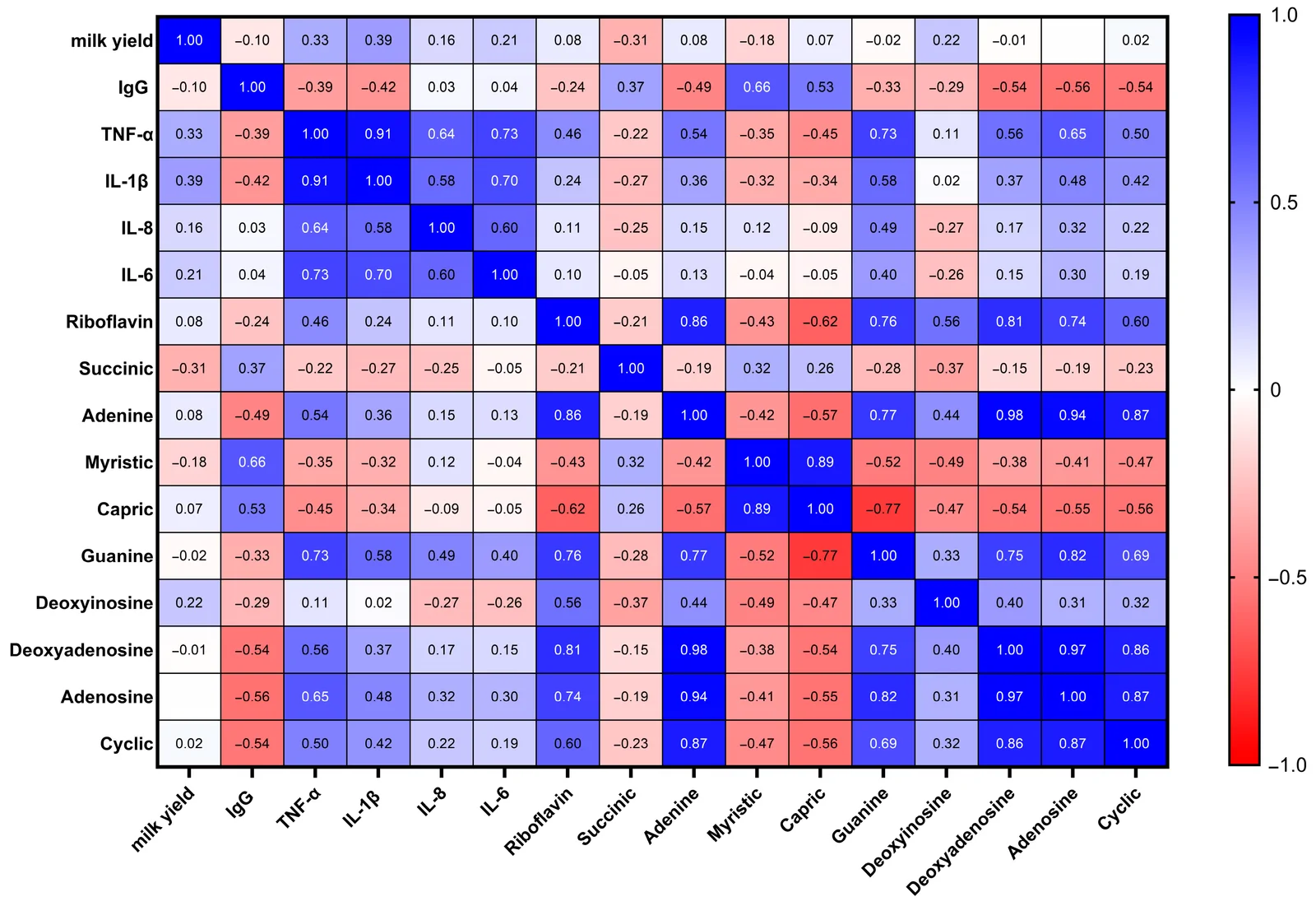
Pearson correlation heatmap between the 10 significant differential metabolites and matched phenotypic traits. The heatmap shows Pearson correlation coefficients between milk yield, plasma immune/inflammatory indicators (IgG, TNF-α, IL-1β, IL-8, and IL-6), and the 10 significant differential metabolites identified in rumen fluid metabolomic analysis, including riboflavin, succinic acid, adenine, myristic acid, capric acid, guanine, deoxyinosine, deoxyadenosine, adenosine, and cyclic AMP. Red indicates a negative correlation and blue indicates a positive correlation, with color intensity corresponding to the strength of the correlation. The values shown in each cell represent Pearson’s r.

Metabolomic analysis was based on 6 biological replicates per treatment collected across the 3 experimental periods. Rumen fluid metabolomic analysis detected 645 metabolite features, and the differential metabolite heatmap showed distinct clustering patterns among the three dietary groups (Figure 2A). KEGG enrichment analysis showed that these differential metabolites were mainly enriched in purine metabolism, ABC transporters, regulation of lipolysis in adipocytes, platelet activation, insulin signaling, MAPK signaling, chemokine signaling, and fatty acid biosynthesis (Figure 2B; Table 11). The OPLS-DA score plot also showed a clear separation among the WB, PKM, and FPKM groups (Figure 2C), and the permutation test indicated that the model was stable and not obviously overfitted, as shown by the negative Q^2^ intercept (Figure 2D). Ten metabolites differed significantly among groups, including riboflavin, succinic acid, adenine, myristic acid, guanine, capric acid, deoxyinosine, deoxyadenosine, adenosine, and cyclic AMP (*p* < 0.05; Table 12). Relative to the WB group, PKM and FPKM showed higher succinic acid, myristic acid, and capric acid, together with lower riboflavin, adenine, and guanine. Compared with PKM, FPKM showed higher riboflavin, adenine, and capric acid, whereas succinic acid, myristic acid, and guanine were lower (Table 12). Correlation analysis further showed that several differential metabolites were associated with matched phenotypic traits (Figure 3). Overall, the correlations between differential metabolites and milk yield were weak. In contrast, clearer associations were observed with immune and inflammatory indicators. Guanine, deoxyadenosine, adenosine, and cyclic AMP were positively correlated with TNF-α and also showed positive correlations with IL-1β, IL-8, and IL-6. By contrast, myristic acid and capric acid were positively correlated with IgG. 

## 4. Discussion

### 4.1. Nutrient Composition

Differences in the chemical composition of WB, PKM, and FPKM resulted in distinct dietary formulations. FPKM was dried after fermentation, which contributed to its higher DM content. During fermentation, enzymes secreted by microorganisms can degrade high-molecular-weight proteins, structural carbohydrates, anti-nutritional factors, and lipids [2,29,30], thereby reducing NDICP, ADICP, NDF, ADF, ADL, and β-mannan, while increasing more readily degradable components such as SP and NPN [31]. It should also be noted that FPKM in the present study was produced from PKM supplemented with wheat middlings and soybean molasses before fermentation. Therefore, the differences observed between PKM and FPKM should be interpreted as the combined consequence of substrate conditioning and microbial biotransformation, rather than as the isolated effect of fermentation alone. Even so, the marked reductions in fiber fractions and β-mannan, together with the increases in soluble nitrogen components, suggest that the changes in FPKM were not due simply to ingredient dilution, but were largely associated with microbial degradation during fermentation. This interpretation is consistent with previous studies showing that solid-state fermentation of PKM can markedly reduce fiber fractions while increasing soluble protein- and peptide-related components [2]. The improved nutrient profile of FPKM was reflected in its higher effective ruminal degradability, which would favor nutrient availability for rumen microbes and the host [32]. Correspondingly, the increase in readily degradable components was accompanied by a reduction in the slowly degradable fraction (b). The relatively higher ash content in FPKM may be related to the concentration effect during fermentation, as minerals are less utilized by microorganisms than organic nutrients [33]. In addition, the use of wheat middlings as a supplementary carbon source may partly explain the higher starch content in FPKM. The present study also showed that β-mannan in PKM could be effectively reduced during both solid-state fermentation and ruminal digestion. However, incomplete degradation may still allow a proportion of β-mannan to reach the lower gut, which may help explain why IL-8 in the FPKM group remained slightly higher than that in the WB group.

### 4.2. Lactation Performance

Differences in feed intake (DMI) and milk production among the groups may be related to variations in the feeding and digestive efficiency of cows when consuming the three different rations. Overall, DMI is a key factor affecting production performance and rumen fermentation [34]. Specifically, the increase in DMI when FPKM replaces WB indicates that fermentation improves feed palatability, possibly due to the production of aromatic compounds during fermentation [35], thereby promoting corresponding changes in CP, NDF, and ADF intake. At the same time, fermentation alters nutrient structure by promoting the degradation of β-mannan, structural carbohydrates, and protein fractions, thereby improving nutrient accessibility for rumen microorganisms and ultimately enhancing nutrient utilization. After fermentation, the FPKM product was dried, milled, and sieved to improve particle-size uniformity before being incorporated into the experimental diets. The improvement in nutrient digestibility was also associated with the higher ED of FPKM, indicating a greater proportion of nutrients available for ruminal degradation and microbial utilization. This increased substrate availability may have supported MCP synthesis and contributed to the higher apparent total-tract digestibility of CP, NDF, and ADF [36]. The enhanced nutrient supply may further support milk synthesis, as reflected by the higher milk yield observed in the FPKM group.

Differences among the different ration groups also confirm the above mechanism. The increased feed intake in the FPKM group may be related to the lower NDF and peNDF content in its ration, as an increase in dietary NDF can increase rumen fill and inhibit feeding [37]; at the same time, the improved physical uniformity of FPKM after processing may have contributed to more consistent nutrient availability during feeding. In contrast, the PKM group, with its high fiber content and low degradability, may result in both lower feed intake and milk production. Although there is little difference in structural carbohydrates among the three ration groups under isocaloric and isonitrogenous formulations, the aromatic flavor produced by FPKM fermentation may improve palatability, thereby stimulating feeding, which further illustrates the positive impact of fermentation on feeding behavior.

Ruminal pH did not differ among treatments. Thus, the higher concentrations of fermentation products in the FPKM group were not accompanied by detectable ruminal acidification. The higher NH_3_-N concentration in this group was consistent with greater availability of degradable nitrogen from dietary CP and NPN, which can be converted to ammonia during ruminal fermentation [38]. MCP concentration also increased, indicating that sufficient fermentable energy was available for microbial growth and microbial protein synthesis [39,40].

The altered VFA profile may reflect improved substrate availability and microbial fermentation efficiency following fermentation treatment. Increased availability of fermentable carbohydrates and degradable nutrients can enhance microbial activity and promote the conversion of dietary substrates into VFA [34]. However, the unchanged acetate-to-propionate ratio suggests that FPKM mainly enhanced the overall fermentation capacity rather than causing a major shift in the predominant fermentation pathway [41].

The altered fermentation pattern may also be related to the functional characteristics of the rumen microbial community. *Prevotella* possesses broad metabolic capabilities for utilizing carbohydrates and proteins and is associated with succinate- and propionate-related fermentation pathways [42,43,44]. Therefore, changes in this bacterial group may contribute to variations in propionate-associated metabolism. Fibrolytic microorganisms, including *Fibrobacterota* and *Fibrobacter*, participate in the degradation of cellulose and other structural carbohydrates [45,46]. Their responses may reflect microbial adaptation to differences in dietary fiber characteristics and substrate accessibility.

The FPKM group also had the highest fecal total VFA concentration and acetate molar proportion. Fecal VFA mainly reflects the fermentation of residual substrates in the hindgut after fermentation and VFA absorption have occurred in the preceding gastrointestinal compartments. The higher fecal VFA concentration may have been partly related to the greater DMI of cows fed FPKM and the resulting increase in substrate flow through the gastrointestinal tract.

However, fecal VFA concentration is not a direct measure of total hindgut VFA production. It is also affected by VFA absorption, digesta passage, and fecal water content. In addition, the microbial analysis in this study was based on rumen-fluid samples rather than fecal samples. The fecal fermentation results therefore could not be directly attributed to changes in individual rumen bacterial taxa.

The elevated ruminal propionate concentration in the FPKM group is a key mechanistic link, as propionate is the primary precursor for hepatic gluconeogenesis [47], which in turn supplies the glucose necessary for lactose synthesis, the main driver of milk volume. In this study, cows fed FPKM had both the highest ruminal propionate concentration and the highest ECM yield. The greater propionate supply may have supported hepatic glucose production and lactose synthesis, contributing to the increase in milk yield. ECM is particularly informative in this context because it adjusts milk output for fat and protein yields. Thus, the higher ECM observed in the FPKM group reflected the combined increases in milk yield, milk fat yield, and milk protein yield rather than an increase in milk volume alone. The higher MCP concentration may also have increased the supply of microbial amino acids available for milk protein synthesis [48,49]. However, ECM/DMI did not differ among treatments, indicating that FPKM increased total energy-corrected milk output without improving ECM-based feed efficiency. Therefore, the higher MCP content in the FPKM and WB groups may be one of the reasons for their higher milk yield and milk protein content. Acetate absorbed from the rumen and β-hydroxybutyrate formed from ruminal butyrate during metabolism by the rumen epithelium are important precursors for de novo fatty acid synthesis in the mammary gland [50]. In the present study, the higher absolute acetate concentration in the FPKM group was consistent with its greater milk fat yield; however, the concurrent increases in propionate concentration and milk yield may have diluted the concentration of fat in milk. In contrast, the PKM group had the highest acetate molar proportion and the lowest milk yield, which may partly explain its higher milk fat percentage despite its lower milk fat yield. Because ruminal butyrate concentration and the acetate-to-propionate ratio did not differ among treatments, the observed response likely reflected the combined effects of acetate supply, milk-volume dilution, and dietary EE rather than the effect of a single ruminal fermentation parameter. In addition, dietary protein intake affects the content of MUN [51]; the higher MUN in the FPKM group may be due to the higher rumen degradable protein (RDP) content, as excess RDP is converted into urea and excreted in milk [52]. The lower RDP/CP ratio in the WB diet may be one mechanism for its lower MCP content compared with FPKM group.

### 4.3. Systemic Inflammation and Immune Response

The elevated levels of pro-inflammatory cytokines (TNF-α, IL-8) in the plasma of cows fed the PKM diet were associated with the high dietary concentration of β-mannan. This correlation is consistent with the established literature wherein β-mannan has been shown to be a potent immune modulator capable of activating pattern recognition receptors, which can lead to the release of inflammatory cytokines [53]. Thus, we hypothesize that the residual β-mannan from PKM, which was not fully degraded in the rumen, may have reached the lower gut and potentially elicited a similar low-grade, systemic inflammatory response. This hypothesized immune activation could represent a metabolic drain, diverting nutrients away from production and providing a plausible explanation for the reduced performance in the PKM group. In contrast, the effective reduction in β-mannan through solid-state fermentation in FPKM likely mitigated this potential trigger, resulting in an inflammatory profile comparable to the WB control group.

### 4.4. Rumen Microbiome

The ruminal microbial shifts observed in this study represent the community’s response to dietary interventions within the 28-day experimental periods. Although the Latin square design with a 21-day adaptation phase helped control individual- and period-related variation at the statistical level, we acknowledge that the rumen ecosystem may exhibit inertia and that a longer duration could be required to reach a fully stabilized state. Accordingly, our results should be interpreted as reflecting short-term or transitional microbial dynamics elicited by diets. In addition, although the replicated 3 × 3 Latin square design helped control variation associated with individual cows and experimental periods, the microbiota and metabolomics analyses were based on 6 biological replicates per treatment accumulated across the 3 experimental periods. This sample size was sufficient to reveal major treatment-related microbial and metabolic shifts, but the statistical power to detect relatively small differences may have been limited. Therefore, the omics findings should be interpreted as exploratory mechanistic evidence and considered together with the production performance, rumen fermentation, digestibility, and plasma inflammatory data. Importantly, under an identical time frame, the significant differences among the WB, PKM, and FPKM groups indicate that these feed sources exert distinct effects on the rumen microenvironment. Overall, dietary interventions markedly altered rumen microbial community structure, underscoring the ecosystem’s capacity to adapt to substrates with different physicochemical characteristics. The rumen microbial community responds dynamically to differences in dietary substrate characteristics. Fibrolytic microorganisms, including *Ruminococcus* and members of *Fibrobacterota*, play important roles in the degradation of cellulose and other structural carbohydrates [45,46,54,55]. Their distribution among diets may reflect adaptation to differences in fiber composition and accessibility. Although changes in individual fibrolytic taxa may influence fiber utilization, the functional redundancy within the rumen ecosystem allows different microbial groups to perform overlapping roles in carbohydrate degradation. Therefore, changes in the abundance of a single bacterial taxon do not necessarily indicate changes in overall fiber-degrading capacity.

Carbohydrate- and protein-utilizing bacteria may also contribute to differences in ruminal fermentation patterns. *Prevotella* possesses broad metabolic capabilities for utilizing polysaccharides and proteins and has been associated with succinate and propionate production pathways [42,43]. Therefore, the altered abundance of *Prevotella* may contribute to differences in propionate-associated fermentation. Similarly, changes in fiber-degrading bacterial populations may be related to variations in acetate production because acetate is a major end product of structural carbohydrate fermentation.

The *Saccharibacteria* is also involved in the carbohydrate fermentation process [44], and its higher abundance in PKM group may be related to the utilization of mannose released from β-mannan degradation; its metabolic products have regulatory effects on rumen microecology and microbial interactions [56]. Its enrichment in the PKM group may therefore reflect a less efficient primary use of fiber-associated substrates, leaving more intermediate products available for secondary fermenters. However, the PKM group had lower total VFA, acetate, and propionate concentrations than the FPKM group. Although its acetate molar proportion was higher, the absolute acetate concentration remained lower, suggesting a difference in the VFA profile rather than greater acetate production. Some *Segatella* strains may also play a role in the fermentation process—its higher abundance in PKM group may be due to its ability to utilize fermentation by-products, but its specific function in rumen needs further study.

In environments with limited nutritional resources, there may be substrate competition between functionally similar bacteria [57]. For example, *Ruminococcus*, as an important cellulolytic bacterium, can decompose cellulose and provide nutrients for other microorganisms [45]; while bacteria in the phylum *Bacillota* have diverse metabolic functions, participating in various pathways such as starch decomposition and protein metabolism [44]. In the FPKM treatment group, *Bacillota* may be competitively inhibited by other functionally similar bacterial communities, leading to a lower relative abundance. Nevertheless, the FPKM group had the highest total VFA, acetate, and propionate concentrations, indicating that the decrease in *Bacillota* did not reduce overall ruminal fermentation and may have been compensated for by other carbohydrate- and fiber-utilizing taxa. This competitive exclusion reflects the dynamic balance of the rumen microbial ecosystem and its adaptation to dietary changes. Taken together, these microbial changes suggest that FPKM supported a rumen community structure more compatible with efficient substrate conversion, enhanced fermentation output, and a lower inflammatory burden than unfermented PKM.

### 4.5. Microbial Metabolites and KEGG Pathway Analysis

The metabolomics data provide a functional readout of the microbial restructuring observed in the FPKM group and help explain how these taxonomic changes may be linked to rumen fermentation efficiency, nutrient metabolism, and inflammatory status. The alterations in purine metabolism (e.g., adenine, guanine) are indicative of shifts in microbial growth, turnover, and community dynamics, serving as a metabolic fingerprint of the microbiome’s response to the different diets. The higher levels of succinic acid, a key intermediate in the propionate production pathway [58], in the PKM and FPKM groups further corroborate the functional potential for enhanced propionate synthesis observed with these diets. When considered together with the enrichment of *Prevotella* and the higher ruminal propionate concentration in the FPKM group, these data suggest that FPKM promoted a rumen environment with greater potential for glucogenic fermentation. Compared to the WB group, the levels of succinic acid, myristic acid, and capric acid were higher in the PKM and FPKM groups, indicating that these two diets may regulate production performance by affecting energy metabolism pathways. Among them, succinic acid participates in cellular energy processes [58]; myristic acid and capric acid, as available fatty acids, are not only energy sources but may also affect inflammatory responses by regulating the immune system [59]. It is particularly noteworthy that in the FPKM treatment group, riboflavin, adenine, capric acid, deoxyribonucleoside, and deoxyadenosine all showed an upregulation trend. In addition to participating in energy metabolism, riboflavin can also regulate the gut microbiota and alleviate inflammation [60]; adenine, deoxyribonucleoside, and deoxyadenosine, as purine metabolism-related substances, participate in cellular energy signaling and nucleic acid metabolism [58]; and adenosine can also exert anti-inflammatory effects by binding to adenosine receptors [61]. Compared with PKM, the increase in riboflavin and adenine and the decrease in succinic acid and guanine in the FPKM group suggest a shift toward a more balanced microbial metabolic state, which may be associated with improved fermentation efficiency and reduced inflammatory pressure. These changes indicate that FPKM treatment may promote efficient energy utilization, optimize nucleotide metabolism, and maintain inflammatory balance by regulating microbial composition and metabolic networks. Correlation analysis further supported the functional relevance of these metabolite changes. Overall, the differential metabolites showed weak correlations with milk yield, whereas clearer relationships were observed with inflammatory and immune indicators. In particular, guanine, deoxyadenosine, adenosine, and cyclic AMP were positively correlated with TNF-α and also tended to be positively associated with IL-1β, IL-8, and IL-6. By contrast, myristic acid and capric acid were positively associated with IgG and negatively associated with TNF-α and IL-1β. These results suggest that the metabolite remodeling associated with FPKM was related more clearly to host inflammatory and immune responses than directly to milk yield. Thus, the advantage of FPKM over PKM appears to extend beyond simple changes in ingredient composition and to include a more favorable rumen functional state, characterized by coordinated changes in microbial community structure, fermentation pattern, and metabolite output. In summary, the transition from WB to FPKM in the diet led to a reshaping of the rumen microbial community, which is further reflected at the metabolome level, including changes in purine metabolites and the accumulation of key metabolic intermediates. These changes culminate in an optimized fermentation profile characterized by higher propionate production. This enhanced ruminal function, together with the alleviation of systemic inflammation, may help explain why the FPKM group showed more favorable responses than the PKM group and generally comparable performance to the WB group. 

### 4.6. Feeding Value and Application Potential of FPKM in Lactating Dairy Cows

Under the conditions of this study, fermentation improved the feeding value of PKM, and FPKM successfully replaced the wheat bran included at 5.86% of dietary DM without adversely affecting lactation performance. This application may be particularly relevant when wheat bran is in short supply or its price increases, especially in palm-oil-producing regions where PKM is readily available. Under such circumstances, FPKM could reduce reliance on conventional feed ingredients, broaden the range of feed resources available for ration formulation, and promote the local utilization of palm-processing by-products.

The economic value of FPKM, however, depends on more than the purchase price of PKM. The costs associated with microbial inoculation, fermentation management, drying, storage, transportation, and quality control must also be considered. Producing FPKM close to the source of PKM may reduce transportation and storage costs, whereas energy-intensive drying or long-distance transport could offset part of this advantage. Because the present study did not include a formal cost–benefit analysis, it cannot be concluded that FPKM would reduce feeding costs under all production conditions. Longer-term commercial trials integrating animal performance, product consistency, and complete processing costs are needed to determine its economic feasibility during routine production and periods of feed shortage.

## 5. Conclusions

In the present study, the FPKM product prepared from PKM with supplementary substrates through solid-state fermentation showed improved nutritional characteristics compared with unfermented PKM. When used to completely replace wheat bran or PKM in the diets of lactating Holstein cows, FPKM was associated with more favorable responses than PKM in dry matter intake, milk production, nutrient digestibility, ruminal fermentation, and some inflammation-related indicators, while showing generally comparable responses to WB for many parameters. The study further showed that FPKM altered the rumen microbiome and metabolome. Taken together, these findings suggest that FPKM may serve as a nutritionally improved form of PKM and can be considered as a complete replacement for wheat bran or PKM in lactating Holstein cow diets when diets are properly balanced for energy and protein. This application may be particularly relevant when wheat bran supply or price is unstable, as FPKM could increase flexibility in dairy feed formulation and support more efficient utilization of palm kernel meal as an agricultural by-product. Further studies are still needed to evaluate the long-term effects of FPKM on cow health, reproductive performance, milk fatty acid profiles, and economic feasibility under commercial production conditions.

## Figures and Tables

**Table 1:** The dietary components and chemical compositions added separately consisted of wheat bran (WB), palm kernel meal (PKM), and fermented palm kernel meal (FPKM) ^1^.

Items	WB (DM %)	PKM (DM %)	FPKM (DM %)
**Ingredient**			
Alfalfa hay	15.87	15.87	15.87
Corn silage	37	37	37
Corn starch	7.75	7.75	7.75
Flaked corn	9.47	9.47	9.47
Soybean meal	15.07	15.07	15.07
DDGS	3.21	3.21	3.21
Wheat bran	5.86	0	0
PKM	0	5.86	0
FPKM	0	0	5.86
Sugar beet pellets	2	2	2
Sodium bicarbonate	1.01	1.01	1.01
Calcium hydrogen phosphate	0.35	0.35	0.35
Limestone powder	1.31	1.31	1.31
Salt	0.44	0.44	0.44
Magnesium oxide	0.22	0.22	0.22
1% premix ^2^	0.44	0.44	0.44
Total	100	100	100
**Chemical composition**			
CP %	16.37	16.36	16.30
RDP/CP %	62.18	60.86	63.7
NE_L_ Mcal	1.74	1.73	1.74
NDF %	30.68	31.96	30.41
ADF %	19.21	20.44	19.71
NFC %	41.22	40.33	41.02
peNDF %	23.39	24.73	23.09
Starch %	27.53	26.29	26.47
EE %	5.66	5.85	5.80

Note: DDGS, distillers dried grains with solubles; NE_L_ was estimated according to Cornell–Penn–Miner dairy (CPM Dairy, v.3.0.10) [16]. DM, dry matter; NEL, net energy for lactating cow; CP, crude protein; RDP, rumen degradable protein; ADF, acid detergent fiber; NDF, neutral detergent fiber; NFC, non-fibrous carbohydrate; peNDF, physically effective neutral detergent fiber; pef, physical effectiveness factor. peNDF (% of DM) = NDF (% of DM) × pef; EE, Ether Extract. ^1^ WB, A basal diet containing wheat bran; PKM, 100% replacement of wheat bran with palm kernel meal; FPKM, 100% replacement of wheat bran with fermented palm kernel meal. ^2^ Contained per kilogram of premix: Ca 142.5 g, P 54.0 g, Mg 49.3 g, Na 106.4 g, Cl 29.5 g, K 500 mg, S 3.7 g, Co 50 mg, Cu 3.0 g, Fe 5.0 g, I 200 mg, Mn 3.8 g, Se 95 mg, Zn 14.5 g, vitamin A 1,200,000 IU, vitamin D 350,000 IU, and vitamin E 6500 IU.

**Table 2:** The conventional nutrients of wheat bran (WB), palm kernel meal (PKM), and fermented palm kernel meal (FPKM).

Items	WB	PKM	FPKM
DM	88.75	90.89	91.72
CP (DM %)	17.00	16.739	16.62
Small peptide (CP %)	2.14	4.99	14.87
NDF (DM %)	44.00	65.80	39.39
ADF (DM %)	15.00	36.08	23.51
NDICP (DM %)	2.89	11.80	7.02
ADICP (DM %)	0.68	3.18	1.59
ADL (DM %)	3.52	6.15	5.07
Ash (DM %)	5.80	5.21	5.76
EE (DM %)	4.50	7.71	6.85
SP (DM %)	6.97	1.78	4.37
NPN (DM %)	3.09	0.25	4.20
Starch (DM %)	20.32	0.6	3.6
β-mannan (DM %) ^1^	3.5	30.0	10.1

Note: DM, dry matter; CP, crude protein; NDF, neutral detergent fiber; ADF, acid detergent fiber; NDICP, neutral detergent insoluble crude protein; ADICP, acid detergent insoluble crude protein; ADL, acid detergent Lignin; EE, ether extract; SP, soluble protein; NPN, non-protein nitrogen; WB, wheat bran; PKM, palm kernel meal; FPCM, fermented palm kernel meal. ^1^ The β-mannan content was analyzed according to the method specified in GB/T 6006-2024 [17].

**Table 3:** Ruminal degradation parameters of palm kernel meal (PKM), and fermented palm kernel meal (FPKM).

Items ^1^	Treatment		SEM	*p*-Value
	PKM	FPKM		
**DM**				
*a*/%	15.43	46.00	0.252	<0.001
*b*/%	84.57	54.00	0.252	<0.001
*c*/(%/h)	1.23	1.62	0.143	0.130
*ED*/%	29.80	57.48	1.02	<0.001
**CP**				
*a*/%	21.25	50.03	0.623	<0.001
*b*/%	78.75	49.97	0.623	<0.001
*c*/(%/h)	0.99	1.01	0.0887	0.900
*ED*/%	32.41	57.20	0.561	<0.001
**NDF**				
*a*/%	1.30	11.41	0.394	<0.001
*b*/%	98.70	88.59	0.394	<0.001
*c*/(%/h)	1.16	1.52	0.107	0.072
*ED*/%	17.27	29.33	1.10	0.002
**ADF**				
*a*/%	1.91	8.17	0.532	0.001
*b*/%	98.09	91.83	0.532	0.001
*c*/(%/h)	1.66	1.85	0.101	0.250
*ED*/%	23.15	29.82	0.832	0.005

Note: Abbreviation: DM, dry matter; CP, crude protein, NDF, neutral detergent fiber; ADF, acid detergent fiber; PKM, palm kernel meal; FPKM, fermented palm kernel meal; SEM, standard error of means. Significant difference, *p* < 0.05. ^1^ *a*, rapidly degradable fraction; *b*, insoluble but potentially degradable fraction; *c*, fractional degradation rate of fraction *b* (%/h; converted to h^−1^ for the calculation of *ED*); *ED*, effective ruminal degradability; *k_p_*, fractional ruminal passage rate, set at 0.06 h^−1^ according to Kolver et al. [27].

**Table 4:** β-mannan content in the rumen after 24-h and 48-h degradation of palm kernel meal (PKM) and fermented palm kernel meal (FPKM).

β-Mannan %	PKM	FPKM	SEM	*p*-Value
24 h %	22.30	9.30	0.995	<0.001
48 h %	16.48	8.48	0.262	<0.001

Note: PKM, palm kernel meal; FPKM, fermented palm kernel meal; SEM, standard error of means. Significant difference, *p* < 0.05.

**Table 5:** Milk production, milk composition, and feed efficiency in lactating Holstein cows fed diets containing WB, PKM, or FPKM.

Items	Treatment			SEM	*p*-Value
	WB	PKM	FPKM		
DMI	23.67 ^b^	22.73 ^c^	24.93 ^a^	0.817	<0.001
**Production, kg/d**					
Milk	30.56 ^b^	29.17 ^c^	32.03 ^a^	0.562	0.002
4% FCM ^1^	29.9 ^b^	28.8 ^b^	31.2 ^a^	0.595	0.005
ECM ^2^	33.0 ^b^	31.5 ^c^	34.4 ^a^	0.692	0.002
Lactose	1.447 ^b^	1.41 ^b^	1.55 ^a^	0.0644	0.017
Fat	1.18 ^ab^	1.14 ^b^	1.23 ^a^	0.0246	0.010
Protein	1.02 ^a^	0.93 ^b^	1.06 ^a^	0.0255	<0.001
**Composition**					
Lactose, %	4.73	4.84	4.84	0.135	0.440
Fat, %	3.85 ^b^	3.91 ^a^	3.84 ^b^	0.0146	<0.001
Protein, %	3.34 ^a^	3.20 ^b^	3.30 ^a^	0.0327	0.002
SCC, ×10^3^ cell/mL	176.25	174.79	179.44	16.6	0.980
MUN, mg/dL	13.97 ^b^	13.53 ^c^	15.00 ^a^	0.235	0.001
**Feed efficiency**					
ECM/DMI	1.42	1.40	1.38	0.0658	0.590

Note: WB, A basal diet containing wheat bran; PKM, 100% replacement of wheat bran with palm kernel meal; FPKM, 100% replacement of wheat bran with fermented palm kernel meal; DMI, dry matter intake; FCM, fat-corrected milk; ECM, energy-corrected milk; SCC, somatic cell count; MUN, milk urea nitrogen; SEM, standard error of means. Significant difference, *p* < 0.05. Different superscripts per row define significant difference. Data were obtained from all cows, yielding 12 cow–period observations per treatment across the three experimental periods (4 cows per treatment per period; *n* = 12). ^1^ 4% FCM = 0.4 × milk yield + 15 × fat yield. ^2^ ECM = 0.3246 × milk yield + 13.86 × milk fat yield + 7.04 × milk protein yield [28].

**Table 6:** Ruminal and fecal fermentation parameters in lactating Holstein cows fed diets containing WB, PKM, or FPKM.

Items	Treatment			SEM	*p*-Value
	WB	PKM	FPKM		
**Ruminal fermentation parameters**					
pH	6.28	6.42	6.24	0.0574	0.140
NH_3_-N, mg/dL	18.15 ^b^	16.62 ^c^	19.49 ^a^	0.882	0.036
MCP, g/d	1757.83 ^b^	1675.63 ^c^	1838.66 ^a^	25.6	0.002
TVFA, mmol/L	94.53 ^b^	91.95 ^c^	98.29 ^a^	1.80	<0.001
Acetate, mmol/L	56.43 ^b^	55.68 ^c^	58.39 ^a^	0.731	<0.001
Propionate, mmol/L	24.00 ^b^	23.64 ^c^	24.50 ^a^	1.10	0.002
Isobutyric acid, mmol/L	0.89	0.93	0.74	0.203	0.410
Butyrate, mmol/L	10.35	9.24	11.74	1.40	0.560
Isovalerate, mmol/L	1.09	0.94	1.14	0.177	0.180
Valerate, mmol/L	1.77	1.52	1.78	0.201	0.240
Acetate: propionate	2.35	2.36	2.38	0.115	0.370
**VFA profile, mol/100 mol**					
Acetate	59.70 ^b^	60.55 ^a^	59.41 ^b^	0.96	0.060
Propionate	25.38	25.71	24.93	1.04	0.580
Isobutyric acid	0.94	1.01	0.75	0.25	0.160
Butyrate	10.95	10.05	11.94	1.52	0.530
Isovalerate	1.15	1.02	1.16	0.0736	0.140
Valerate	1.87	1.65	1.81	0.264	0.380
**Fecal fermentation parameters**					
TVFA, mmol/g of feces	0.09 ^b^	0.08 ^c^	0.10 ^a^	0.00189	0.001
Acetate, mol/100 mol	55.18 ^b^	53.46 ^c^	55.48 ^a^	0.842	0.004
Propionate, mol/100 mol	28.91	28.64	29.63	2.92	0.530
Butyrate mol/100 mol	15.91	17.90	13.89	9.01	0.260

Note: WB, a basal diet containing wheat bran; PKM, 100% replacement of wheat bran with palm kernel meal; FPKM, 100% replacement of wheat bran with fermented palm kernel meal; NH_3_-N, ammonia nitrogen; MCP, microbial crude protein; TVFA, total volatile fatty acids; VFA, volatile fatty acids; SEM, standard error of means. Significant difference, *p* < 0.05. Different superscripts per row define significant difference. Each treatment included six cow–period observations collected across the three experimental periods (2 cows per treatment per period; *n* = 6).

**Table 7:** Nutrient intake and apparent total-tract digestibility in lactating Holstein cows fed diets containing WB, PKM, or FPKM.

Items	Treatment			SEM	*p*-Value
	WB	PKM	FPKM		
**Intake, kg/d**					
CP	3.82 ^b^	3.71 ^c^	4.06 ^a^	0.151	<0.001
NDF	7.16 ^b^	7.26 ^b^	7.58 ^a^	0.274	0.003
ADF	4.48 ^c^	4.64 ^b^	4.91 ^a^	0.174	<0.001
**Digestibility, %**					
DM	71.29 ^a^	68.56 ^b^	70.21 ^a^	0.716	0.033
CP	75.41 ^a^	71.72 ^b^	76.90 ^a^	0.733	0.006
NDF	54.04 ^a^	52.62 ^b^	55.43 ^a^	1.32	<0.001
ADF	35.93 ^a^	32.98 ^b^	36.69 ^a^	1.46	0.013

Note: WB, A basal diet containing wheat bran; PKM, 100% replacement of wheat bran with palm kernel meal; FPKM, 100% replacement of wheat bran with fermented palm kernel meal; DM, dry matter; CP, crude protein; NDF, neutral detergent fiber; ADF, acid detergent fiber; SEM, standard error of means. Significant difference, *p* < 0.05. Different superscripts per row define significant difference. Each treatment included six cow–period observations collected across the three experimental periods (2 cows per treatment per period; *n* = 6).

**Table 8:** Plasma biomarkers in lactating Holstein cows fed diets containing WB, PKM, or FPKM.

Items	Treatment			SEM	*p*-Value
	WB	PKM	FPKM		
NEFA, mmol/L	0.143	0.136	0.161	0.0283	0.760
BHBA, mmol/L	0.746	0.747	0.655	0.0858	0.250
GLU, mmol/L	3.71	3.72	3.54	0.221	0.620
IgA, g/L	2.59	2.57	2.59	0.165	0.990
IgG, g/L	10.64 ^a^	9.48 ^b^	10.52 ^a^	0.452	0.032
IgM, g/L	0.696	0.782	0.751	0.0402	0.330
C3, g/L	3.39	4.26	4.35	0.561	0.400
T-SOD, U/mL	74.49	71.85	79.12	5.66	0.057
GSH-PX, U/mL	144.85	147.8	148.39	8.45	0.720
CAT, U/mL	39.7	41.01	40.58	2.95	0.590
MDA, nmol/mL	3.97	3.67	3.64	0.344	0.390
TNF-α, pg/mL	52.64 ^b^	64.98 ^a^	52.12 ^b^	8.70	0.013
IL-1β, pg/mL	21.18 ^b^	24.14 ^a^	20.53 ^b^	3.35	0.071
IL-8, pg/mL	86.88 ^b^	91.39 ^a^	81.91 ^c^	1.70	0.004
IL-6, pg/mL	124.84 ^b^	128.45 ^a^	122.73 ^c^	1.46	0.078

Note: WB, A basal diet containing wheat bran; PKM, 100% replacement of wheat bran with palm kernel meal; FPKM, 100% replacement of wheat bran with fermented palm kernel meal; NEFA, non-esterified fatty acids; BHBA, β-hydroxybutyrate; GLU, glucose; IgA, immunoglobulin A; IgG, immunoglobulin G; IgM, immunoglobulin M; C3, complement component 3; T-SOD, total superoxide dismutase; GSH-Px, glutathione peroxidase; CAT, catalase; MDA, malondialdehyde; TNF-α, tumor necrosis factor-α; IL-1β, interleukin-1β; IL-6, interleukin-6; IL-8, interleukin-8; SEM, standard error of means. Significant difference, *p* < 0.05. Different superscripts per row define significant difference. Each treatment included six cow–period observations collected across the three experimental periods (2 cows per treatment per period; *n* = 6).

**Table 9:** Relative abundance of the 15 most abundant rumen bacterial phyla in lactating Holstein cows fed diets containing WB, PKM, or FPKM.

Items	Treatment			SEM	*p*-Value
	WB	PKM	FPKM		
*Bacteroidota*	14.88 ^b^	19.89 ^a^	17.97 ^ab^	1.06	0.008
*Bacillota*	11.29 ^a^	9.68 ^ab^	8.19 ^b^	1.07	0.019
*Uroviricota*	1.46	1.18	1.15	1.14	0.410
*Pseudomonadota*	0.69	1.11	0.77	1.20	0.360
*Spirochaetota*	0.81	0.99	0.87	1.10	0.310
*Chytridiomycota*	0.69	0.39	0.38	1.35	0.320
*Ascomycota*	0.59	0.35	0.32	1.35	0.310
*Mucoromycota*	0.57	0.33	0.31	1.35	0.300
*Fibrobacterota*	0.29 ^b^	0.52 ^a^	0.43 ^a^	1.11	0.003
*Basidiomycota*	0.28	0.16	0.15	1.35	0.300
*Zoopagomycota*	0.25	0.14	0.14	1.34	0.300
*Mycoplasmatota*	0.19	0.22	0.19	1.08	0.470
*Actinomycetota*	0.19	0.17	0.16	1.05	0.068
*Verrucomicrobiota*	0.08 ^b^	0.09 ^ab^	0.14 ^a^	1.13	0.030
*Euryarchaeota*	0.11	0.09	0.10	1.10	0.230
*Melainabacteria*	0.08	0.10	0.10	1.16	0.480
*Saccharibacteria*	0.06 ^b^	0.09 ^a^	0.07 ^ab^	1.10	0.045
*Absconditabacteria*	0.04 ^b^	0.09 ^a^	0.07 ^ab^	1.26	0.033
*Blastocladiomycota*	0.07	0.04	0.04	1.32	0.270
*Lentisphaerota*	0.04 ^b^	0.05 ^ab^	0.07 ^a^	1.12	0.024
Others	66.35	63.66	67.07	1.02	0.050

Note: WB, A basal diet containing wheat bran; PKM, 100% replacement of wheat bran with palm kernel meal; FPKM, 100% replacement of wheat bran with fermented palm kernel meal; SEM, standard error of means. Significant difference, *p* < 0.05. Different superscripts per row define significant difference.

**Table 10:** Relative abundance of major rumen bacterial genera in lactating Holstein cows fed diets containing WB, PKM, or FPKM.

Items	Treatment			SEM	*p*-Value
	WB	PKM	FPKM		
*Prevotella*	5.63 ^b^	7.88 ^a^	7.54 ^a^	0.531	0.020
*Xylanibacter*	1.62 ^b^	2.29 ^ab^	2.43 ^a^	0.209	0.034
*Bacteroides*	1.62	1.90	1.77	0.0683	0.980
*Ruminococcus*	1.86 ^a^	1.21 ^ab^	1.06 ^b^	0.183	0.012
*Segatella*	0.78 ^b^	1.40 ^a^	0.93 ^b^	0.112	0.005
*Clostridium*	0.99	1.03	0.83	0.0664	0.100
*Treponema*	0.77	0.93	0.81	0.0819	0.380
*Butyrivibrio*	0.66	0.62	0.64	0.0534	0.870
*Succiniclasticum*	0.60	0.49	0.40	0.0527	0.120
*Selenomonas*	0.57	0.51	0.22	0.219	0.400
*Fibrobacter*	0.29 ^b^	0.52 ^a^	0.45 ^ab^	0.0512	0.047
*Hallella*	0.18 ^b^	0.91 ^a^	0.18 ^b^	0.146	0.025
*Alistipes*	0.42	0.41	0.41	0.0387	0.98
*Phocaeicola*	0.32	0.38	0.35	0.0179	0.170
*Hoylesella*	0.24	0.31	0.24	0.0234	0.160
*Sodaliphilus*	0.24	0.30	0.21	0.0507	0.540
*Eubacterium*	0.27	0.24	0.21	0.0185	0.130
*Parabacteroides*	0.23	0.25	0.23	0.0133	0.440
*Oscillibacter*	0.24	0.20	0.19	0.0358	0.540
*Pseudobutyrivibrio*	0.13	0.13	0.14	0.0124	0.850
Others	82.12 ^a^	78.35 ^b^	80.78 ^ab^	0.839	0.020

Note: WB, A basal diet containing wheat bran; PKM, 100% replacement of wheat bran with palm kernel meal; FPKM, 100% replacement of wheat bran with fermented palm kernel meal; SEM, standard error of means. Significant difference, *p* < 0.05. Different superscripts per row define significant difference.

**Table 11:** Significantly enriched rumen metabolic pathways in lactating Holstein cows fed diets containing WB, PKM, or FPKM.

Pathway_ ID ^1^	Description	Ratio_in_Study	Ratio_in_Pop	*p*_Value	*Q*_Value
ko00230	Purine metabolism	6/23	101/6490	<0.001	<0.001
ko02010	ABC transporters	5/23	138/6490	<0.001	<0.001
ko04923	Regulation of lipolysis in adipocytes	2/23	14/6490	0.0011	0.013
ko04611	Platelet activation	2/23	14/6490	0.0011	0.013
ko04910	Insulin signaling pathway	1/23	4/6490	0.0141	0.057
ko04010	MAPK signaling pathway	1/23	5/6490	0.0176	0.058
ko04062	Chemokine signaling pathway	1/23	5/6490	0.0176	0.058
ko00061	Fatty acid biosynthesis	2/23	58/6490	0.0176	0.058

^1^ ID, identity document.

**Table 12:** Differential rumen metabolites in lactating Holstein cows fed diets containing WB, PKM, or FPKM.

ID ^1^	Metabolite	FC	*p*-Value	VIP ^2^	Up or Down		
					P/W ^3^	F/W ^4^	F/P ^5^
POS_377.1441_213.7234	Riboflavin	2.2808	0.019	1.668	down	down	up
NEG_117.0174_559.8799	Succinic acid	0.5361	0.022	1.388	up	up	down
NEG_134.0454_125.2733	Adenine	3.0125	0.018	1.892	down	down	up
NEG_227.1999_450.2721	Myristic acid	0.3327	<0.001	2.534	up	up	down
NEG_171.1372_337.3545	Capric acid	0.2332	<0.001	2.688	up	up	up
POS_152.0562_91.9687	Guanine	2.2299	0.001	1.608	down	down	down
NEG_251.077_181.0099	Deoxyinosine	2.4823	0.009	2.358	down	down	up
POS_252.1083_194.0631	Deoxyadenosine	2.1905	0.043	1.457	down	down	up
POS_268.1032_191.9326	Adenosine	5.3232	0.034	1.804	down	down	down
NEG_328.0435_115.0316	Cyclic AMP	3.8229	0.005	1.870	down	down	down

^1^ ID, identity document. ^2^ VIP, variable importance in projection. ^3^ P/W, palm kernel meal group vs. wheat bran group. ^4^ F/W, fermented palm kernel meal group vs. wheat bran group. ^5^ F/P, fermented palm kernel meal group vs. palm kernel meal group. Note: The first column is the ID of the metabolite, the second column is the English name of the metabolite, and in the following order: fold of difference, *p* value for univariate analysis, VIP value for OPLS-DA model, and up- and down-regulation.

## Data Availability

The original data presented in this study are openly available in the NCBI Sequence Read Archive (SRA) under accession number PRJNA1297090 and the National Genomics Data Center (NGDC/CNCB) under accession number PRJCA062972.

## References

[1] Nair K.P.P. (2010). Oil palm (*Elaeis guineensis* Jacquin). The Agronomy and Economy of Important Tree Crops of the Developing World.

[2] Qamar H., He R., Li Y., Song M., Deng D., Cui Y., Yu M., Ma X. (2024). Metabolome and metagenome integration unveiled synthesis pathways of novel antioxidant peptides in fermented lignocellulosic biomass of palm kernel meal. Antioxidants.

[3] Kahar P., Rachmadona N., Pangestu R., Palar R., Adi D.T.N., Juanssilfero A.B., Yopi, Manurung I., Hama S., Ogino C. (2022). An integrated biorefinery strategy for the utilization of palm-oil wastes. Bioresour. Technol..

[4] Zhu X., Deng Z., Wang Q., Hao S., Liu P., He S., Li X. (2024). Improvement in palm kernel meal quality by solid-sate fermentation with *Bacillus velezensis*, *Saccharomyces cerevisiae* and *Lactobacillus paracasei*. Fermentation.

[5] Ong W.L., Chan K.L., Suwanto A., Li Z., Ng K.-H., Zhou K. (2024). Hydrolysis of palm kernel meal fibre using a newly isolated *Bacillus subtilis* F6 with high mannanase activity. Bioresour. Bioprocess..

[6] Chen Z., Mense A.L., Brewer L.R., Shi Y.-C. (2024). Wheat bran layers: Composition, structure, fractionation, and potential uses in foods. Crit. Rev. Food Sci. Nutr..

[7] Rosani U., Hernaman I., Hidayat R., Hidayat D. (2024). Use of rice bran as ruminant feed in Indonesia. Int. J. Res. Sci. Innov..

[8] Daniel S., Zeid S., Liao J., Hang S. (2025). Exploring the effect of feeding broiler chickens a diet incorporating unfermented or fermented palm kernel cake: Growth performance, digestibility, biochemical indices, digestive enzyme activity, and mRNA gene expression of nutrient transporters. Ital. J. Anim. Sci..

[9] Żelechowska P., Brzezińska-Błaszczyk E., Różalska S., Agier J., Kozłowska E. (2021). Mannan activates tissue native and IgE-sensitized mast cells to proinflammatory response and chemotaxis in TLR4-dependent manner. J. Leukoc. Biol..

[10] Nguyen T.N.Y., Matangkasombut O., Ritprajak P. (2018). Differential dendritic cell responses to cell wall mannan of *Candida albicans*, *Candida parapsilosis*, and *Candida dubliniensis*. J. Oral Sci..

[11] Wei X., Wang C., Zhao M., Li J., Wu M. (2014). Directed modification of β-mannanase substrate affinity based on rational design. J. Zhejiang Univ. (Agric. Life Sci.).

[12] Lindstad L.J., Lo G., Leivers S., Lu Z., Michalak L., Pereira G.V., Røhr Å.K., Martens E.C., McKee L.S., Louis P. (2021). Human gut *Faecalibacterium prausnitzii* deploys a highly efficient conserved system to cross-feed on β-mannan-derived oligosaccharides. mBio.

[13] Wang T., Li S., Ning J., Li J., Han Y., Yin X., Huang X., Huang F. (2023). Effects of different processing techniques of palm kernel cake on processing quality of pellet feed, nutrient digestibility, and intestinal microbiota of pigs. J. Anim. Sci..

[14] Jiang W., Zhang Y., Cheng H., Hu X., You W., Song E., Hu Z., Jiang F. (2024). Fermented palm kernel cake improves the rumen microbiota and metabolome of beef cattle. Animals.

[15] Mertens D.R. (1997). Creating a system for meeting the fiber requirements of dairy cows. J. Dairy Sci..

[16] Wei Z., Zhang B., Liu J. (2018). Effects of the dietary nonfiber carbohydrate content on lactation performance, rumen fermentation, and nitrogen utilization in mid-lactation dairy cows receiving corn stover. J. Anim. Sci. Biotechnol..

[17] (2024). Determination of Mannan and β-Glucan in Saccharomyces cerevisiae Culture—High Performance Liquid Chromatography.

[18] AOAC International (2000). Official Methods of Analysis of AOAC International.

[19] Van Soest P.J., Robertson J.B., Lewis B.A. (1991). Methods for dietary fiber, neutral detergent fiber, and nonstarch polysaccharides in relation to animal nutrition. J. Dairy Sci..

[20] Licitra G., Hernandez T.M., Van Soest P.J. (1996). Standardization of procedures for nitrogen fractionation of ruminant feeds. Anim. Feed Sci. Technol..

[21] Lee C., Hristov A.N. (2013). Short communication: Evaluation of acid-insoluble ash and indigestible neutral detergent fiber as total-tract digestibility markers in dairy cows fed corn silage-based diets. J. Dairy Sci..

[22] Huhtanen P., Kaustell K., Jaakkola S. (1994). The use of internal markers to predict total digestibility and duodenal flow of nutrients in cattle given six different diets. Anim. Feed Sci. Technol..

[23] Lacey E.K., Harvatine K.J., Dechow C.D. (2020). Short communication: Diet digestibility measured from fecal samples and associations with phenotypic and genetic merit for milk yield and composition. J. Dairy Sci..

[24] Broderick G.A., Kang J.H. (1980). Automated simultaneous determination of ammonia and total amino acids in ruminal fluid and in vitro media. J. Dairy Sci..

[25] Nuez-Ortín W.G., Yu P. (2010). Estimation of ruminal and intestinal digestion profiles, hourly effective degradation ratio and potential N to energy synchronization of co-products from bioethanol processing. J. Sci. Food Agric..

[26] Ørskov E.R., McDonald I. (1979). The estimation of protein degradability in the rumen from incubation measurements weighted according to rate of passage. J. Agric. Sci..

[27] Kolver E.S., Muller L.D., Varga G.A., Cassidy T.J. (1998). Synchronization of ruminal degradation of supplemental carbohydrate with pasture nitrogen in lactating dairy cows. J. Dairy Sci..

[28] Gu F.F., Liang S.L., Wei Z.H., Wang C.P., Liu H.Y., Liu J.X., Wang D.M. (2018). Short communication: Effects of dietary addition of N-carbamoylglutamate on milk composition in mid-lactating dairy cows. J. Dairy Sci..

[29] Zhang S., Huang Z., Li Q., Zheng X., Liu J. (2024). Two-stage solid-state fermentation to increase the nutrient value of corn processing waste and explore its efficacy as a feed protein source. Food Chem. X.

[30] Saravanakumar S., Prabakaran N.N., Ashokkumar R., Jamuna S. (2024). Unlocking the gut’s treasure: Lipase-producing *Bacillus subtilis* probiotic from the intestine of *Microstomus kitt* (lemon sole). Appl. Biochem. Biotechnol..

[31] Chen Q., Yang X., Liu S., Hong P., Zhou C., Zhong S., Zhu C., Chen J., Chen K. (2024). Changes in protein and volatile flavor compounds of low-salt wet-marinated fermented golden pomfret during processing. Food Chem..

[32] Fan G.-J., Chen M.-H., Lee C.-F., Yu B., Lee T.-T. (2022). Effects of rice straw fermented with spent *Pleurotus sajor-caju* mushroom substrates on milking performance in Alpine dairy goats. Anim. Biosci..

[33] Li C., Chen X., Jin Z., Gu Z., Rao J., Chen B. (2021). Physicochemical property changes and aroma differences of fermented yellow pea flours: Role of lactobacilli and fermentation time. Food Funct..

[34] Khorasani G.R., Okine E.K., Kennelly J.J. (2001). Effects of forage source and amount of concentrate on rumen and intestinal digestion of nutrients in late-lactation cows. J. Dairy Sci..

[35] He Y., Liu T., Larsen D.S., Lei Y., Huang M., Zhu L., Daglia M., Xiao X. (2025). Barley fermentation on nutritional constituents: Structural changes and structure–function correlations. Crit. Rev. Food Sci. Nutr..

[36] Wang Y., Li X., Li K., Huang Y., Yang H., Zhu P., Chi Z., Xu Y., Li Q. (2022). Signature of dissolved organic matter and microbial communities based on different oxygen levels response during distillers dried grains with solubles plus sugarcane pith co-fermentations. Bioresour. Technol..

[37] Palmonari A., Federiconi A., Formigoni A. (2024). Animal board invited review: The effect of diet on rumen microbial composition in dairy cows. Animal.

[38] Zeleke A.W., Dimonaco N.J., Lawther K., Lavery A., Ferris C., Moorby J., Huws S.A. (2025). Reducing crude protein content in the diet of lactating dairy cows improved nitrogen-use-efficiency and reduced N excretion in urine, whilst having no obvious effects on the rumen microbiome. J. Anim. Sci. Biotechnol..

[39] Li W., Cui Z., Jiang Y., Aisikaer A., Wu Q., Zhang F., Wang W., Bo Y., Yang H. (2023). Dietary guanidine acetic acid improves ruminal antioxidant capacity and alters rumen fermentation and microflora in rapid-growing lambs. Antioxidants.

[40] Zhou S., Wang Y., Hu Q., Li J., Chen J., Liu X. (2025). Enhancement of nutritional quality of chickpea flour by solid-state fermentation for improvement of in vitro antioxidant activity and protein digestibility. Food Chem..

[41] Liang J., Zhang H., Zhang P., Zhang G., Cai Y., Wang Q., Zhou Z., Ding Y., Zubair M. (2021). Effect of substrate load on anaerobic fermentation of rice straw with rumen liquid as inoculum: Hydrolysis and acidogenesis efficiency, enzymatic activities and rumen bacterial community structure. Waste Manag..

[42] Vishnu Prasoodanan P.K., Sharma A.K., Mahajan S., Dhakan D.B., Maji A., Scaria J., Sharma V.K. (2021). Western and non-western gut microbiomes reveal new roles of *Prevotella* in carbohydrate metabolism and mouth–gut axis. npj Biofilms Microbiomes.

[43] Huws S.A., Creevey C.J., Oyama L.B., Mizrahi I., Denman S.E., Popova M., Muñoz-Tamayo R., Forano E., Waters S.M., Hess M. (2018). Addressing global ruminant agricultural challenges through understanding the rumen microbiome: Past, present, and future. Front. Microbiol..

[44] Liu L., Wu P., Guo A., Yang Y., Chen F., Zhang Q. (2023). Research progress on the regulation of production traits by gastrointestinal microbiota in dairy cows. Front. Vet. Sci..

[45] Zhang Z., Niu X., Li F., Li F., Guo L. (2020). Ruminal cellulolytic bacteria abundance leads to the variation in fatty acids in the rumen digesta and meat of fattening lambs. J. Anim. Sci..

[46] Auer E., Lazuka A., Huguenin-Bizot B., Jehmlich N., Déjean S., Lombard V., Henrissat B., O’Donohue M., Hernandez-Raquet G. (2023). Horizontal metaproteomics and CAZymes analysis of lignocellulolytic microbial consortia selectively enriched from cow rumen and termite gut. ISME Commun..

[47] Sauer F.D., Kramer J.K.G., Cantwell W.J. (1989). Antiketogenic effects of monensin in early lactation. J. Dairy Sci..

[48] Wang M.Z., Jing Y.J., Wang Y.F., Liu S.M., Gao J., Ouyang J.L., Vercoe P. (2019). Effects of unsaturation of long-chain fatty acids on rumen protozoal engulfment and microbial protein recycling in protozoa in vitro. Anim. Prod. Sci..

[49] Zhou X.Q., Zhang Y.D., Zhao M., Zhang T., Zhu D., Bu D.P., Wang J.Q. (2015). Effect of dietary energy source and level on nutrient digestibility, rumen microbial protein synthesis, and milk performance in lactating dairy cows. J. Dairy Sci..

[50] Hassan F., Tang Z., Ebeid H.M., Li M., Peng K., Liang X., Yang C. (2021). Consequences of herbal mixture supplementation on milk performance, ruminal fermentation, and bacterial diversity in water buffaloes. PeerJ.

[51] Zhao X., Zheng N., Zhang Y., Wang J. (2025). The role of milk urea nitrogen in nutritional assessment and its relationship with phenotype of dairy cows: A review. Anim. Nutr..

[52] Mortazavi M., Zandi M.B., Pahlavan R., Nasab M.E., Mulim H.A., de Oliveira H.R. (2025). Genome-wide association analysis based on random regression models for milk urea nitrogen in Iranian Holstein cattle. Dairy Sci. Manag..

[53] de Kivit S., Tobin M.C., Forsyth C.B., Keshavarzian A., Landay A.L. (2014). Regulation of intestinal immune responses through TLR activation: Implications for pro- and prebiotics. Front. Immunol..

[54] Bhagat N.R., Kumar S., Kumari R., Bharti V.K. (2023). A review on rumen anaerobic fungi: Current understanding on carbohydrate fermentation and roughages digestion in ruminants. Appl. Biochem. Microbiol..

[55] Patra A.K., Yu Z. (2015). Essential oils affect populations of some rumen bacteria in vitro as revealed by microarray (RumenBactArray) analysis. Front. Microbiol..

[56] Xu Q., Qiao Q., Gao Y., Hou J., Hu M., Du Y., Zhao K., Li X. (2021). Gut microbiota and their role in health and metabolic disease of dairy cow. Front. Nutr..

[57] Gusmawartati, Sari P.R. (2023). Cellulolytic bacteria as decomposers in dry empty fruit bunches composting. IOP Conf. Ser. Earth Environ. Sci..

[58] Woulfe J., Munoz D.G., Gray D.A., Jinnah H.A., Ivanova A. (2024). Inosine monophosphate dehydrogenase intranuclear inclusions are markers of aging and neuronal stress in the human substantia nigra. Neurobiol. Aging.

[59] Shao P., Sha Y., Liu X., He Y., Wang F., Hu J., Wang J., Li S., Chen X., Yang W. (2024). Supplementation with *Astragalus* root powder promotes rumen microbiota density and metabolome interactions in lambs. Animals.

[60] Brandão T.A.S., Vieira L.A., de Araújo S.S., Nagem R.A.P. (2023). Probing the mechanism of flavin action in the oxidative decarboxylation catalyzed by salicylate hydroxylase. Methods Enzymol..

[61] Yang Y., Liu Y., Wang Y., Chao Y., Zhang J., Jia Y., Tie J., Hu D. (2022). Regulation of SIRT1 and its roles in inflammation. Front. Immunol..

